# CR3 and Dectin-1 Collaborate in Macrophage Cytokine Response through Association on Lipid Rafts and Activation of Syk-JNK-AP-1 Pathway

**DOI:** 10.1371/journal.ppat.1004985

**Published:** 2015-07-01

**Authors:** Juin-Hua Huang, Ching-Yu Lin, Sheng-Yang Wu, Wen-Yu Chen, Ching-Liang Chu, Gordon D. Brown, Chih-Pin Chuu, Betty A. Wu-Hsieh

**Affiliations:** 1 Graduate Institute of Immunology, National Taiwan University College of Medicine, Taipei, Taiwan; 2 Institute of Cellular and System Medicine, National Health Research Institutes, Miaoli, Taiwan; 3 Aberdeen Fungal Group, School of Medical Sciences, Institute of Medical Sciences, University of Aberdeen, Aberdeen, United Kingdom; University of Pittsburgh, UNITED STATES

## Abstract

Collaboration between heterogeneous pattern recognition receptors (PRRs) leading to synergistic coordination of immune response is important for the host to fight against invading pathogens. Although complement receptor 3 (CR3) and Dectin-1 are major PRRs to detect fungi, crosstalk between these two receptors in antifungal immunity is largely undefined. Here we took advantage of *Histoplasma capsulatum* which is known to interact with both CR3 and Dectin-1 and specific particulate ligands to study the collaboration of CR3 and Dectin-1 in macrophage cytokine response. By employing Micro-Western Array (MWA), genetic approach, and pharmacological inhibitors, we demonstrated that CR3 and Dectin-1 act collaboratively to trigger macrophage TNF and IL-6 response through signaling integration at Syk kinase, allowing subsequent enhanced activation of Syk-JNK-AP-1 pathway. Upon engagement, CR3 and Dectin-1 colocalize and form clusters on lipid raft microdomains which serve as a platform facilitating their cooperation in signaling activation and cytokine production. Furthermore, *in vivo* studies showed that CR3 and Dectin-1 cooperatively participate in host defense against disseminated histoplasmosis and instruct adaptive immune response. Taken together, our findings define the mechanism of receptor crosstalk between CR3 and Dectin-1 and demonstrate the importance of their collaboration in host defense against fungal infection.

## Author Summary

The incidence of life-threatening fungal infections is increasing during the last decades. A better understanding of the interactions between fungal pathogen and its host cell is important to the development of new therapeutic strategies against fungal infections. Dimorphic fungus *Histoplasma capsulatum* becomes disseminated and threatens life in immunocompromised individuals. This fungal pathogen utilizes complement receptor 3 (CR3) and Dectin-1, two pattern recognition receptors on the surface of innate immune cells, to induce macrophage cytokine response. In this study, we demonstrated that CR3 and Dectin-1 act collaboratively to induce macrophage TNF and IL-6 response through a mechanism dependent on activation of the Syk-JNK-AP-1 signaling axis. CR3 and Dectin-1 are recruited and form clusters on lipid raft microdomains upon stimulation by *H*. *capsulatum*, leading to activation of their signaling convergence at Syk kinase and induction of subsequent cytokine response. In addition, we showed that CR3 and Dectin-1 cooperatively instruct the adaptive antifungal immunity to defense against *H*. *capsulatum* infection. Our findings define the molecular mechanisms underlying receptor crosstalk between CR3 and Dectin-1 and provide a valuable model for receptor collaboration in the context of host-fungus interactions.

## Introduction

Diseases caused by fungal pathogens have become an important cause of morbidity and mortality over the last decades due to the increasing number of immunocompromised patients [1]. To reveal the cellular and molecular mechanisms of the interaction between host and fungal pathogens will be helpful for the development of new therapeutic strategies. Innate immune cells recognize pathogen-associated molecular patterns (PAMPs) on fungi through pattern recognition receptors (PRRs) [2, 3]. The fungal cell wall is composed predominantly of glucans, chitin, mannose and other covalently-linked proteins with the composition varies between species and even between the different strains and morphological forms of the same species [4, 5]. Since a single pathogen is composed of multiple PAMPs, innate immune cells are likely to simultaneously or sequentially utilize a complex set of PRRs to interact with a specific pathogen. The coordination between PRRs leading to activation or inhibition of downstream signaling is referred to as “receptor crosstalk” [6].

PRR collaboration is known to be important for the host to control invading pathogens. It has been reported that Dectin-1 functions synergistically with TLR2 in amplifying innate cell cytokine response when stimulated with their respective ligands [7–10]. Collaboration between Dectin-1 and TLR2 is mediated by the activation of both Dectin-1/Syk and TLR/Myd88 pathways which results in increased NF-κB activity [7, 9]. Receptor crosstalk between C-type lectin receptors (CLRs) has also been reported. Dectin-1 collaboration with SIGNR1 or with Dectin-2 is largely dependent on the activation of Syk kinase to enhance immune responses against *Candida albicans* [11, 12]. Stimulation with *C*. *albicans* induces colocolization and physical association of Dectin-1 and SIGNR1 whose collaboration results in macrophage oxidative response [11]. Aside from collaborating with TLRs and CLRs, Dectin-1 is also known to collaborate with CR3 in macrophage cytokine response to *H*. *capsulatum* in a Syk kinase-dependent manner [13]. However, the coordinated mechanisms in CR3 and Dectin-1 collaboration leading to cytokine production are undefined.

CR3 (Mac-1, α_M_β_2_, or CD11b/CD18) is the principal β2 integrin known to contribute to fungal recognition in innate immune cells [3]. CR3 contains two ligand binding sites, I domain and lectin-like domain, which bind to protein ligands (such as iC3b, ICAM-1, and fibronectin) and β-glucan, respectively [14]. CR3 is an enigmatic receptor which transduces diverse and distinct signaling upon engagement with different ligands [15, 16]. Activation of CR3 is mediated by conformation change and regulated by inside-out and outside-in signals [17]. The inside-out signaling to activate CR3 can be initiated from other receptors, such as TLRs and Dectin-1 [18, 19]. Engagement of CR3 also elicits outside-in signaling which activates innate immune effector functions, such as phagocytosis, cytotoxic killing, and cytokine production [17]. Despite many studies revealing the molecular mechanisms regulating CR3 phagocytosis [20], the signaling pathway(s) responsible for its cytokine response is yet to be addressed.

In contrast to CR3, the understanding of Dectin-1 signaling is growing during the last decade. Dectin-1 engagement induces the phosphorylation of its intracellular ITAM-like motif leading to the recruitment and activation of Syk [21]. Syk facilitates the phosphorylation of PLCγ2, allowing subsequent activation of MAPKs, AP-1 and NFAT and the assembly of Card9-Bcl10-Malt1 signalsome which mediates the canonical NF-κB activation [22–24]. A recent study also showed that Card9 bridges the interaction between Ras-GRF-1 and H-Ras, leading to downstream ERK activation upon Dectin-1 ligation [25]. In addition, Dectin-1 triggers Syk-independent Raf-1 activation through which antagonizes Syk-induced noncanonical NF-κB activation [26]. The requirement for Syk and the use of Card9 in Dectin-1 signaling differs in different macrophage and dendritic cell (DC) populations [27, 28]. Thus, signaling pathway downstream of Dectin-1 and signaling crosstalk between Dectin-1 and heterologous PRRs can very well be cell-type specific.

Here we used *H*. *capsulatum* and particulate ligands to study the molecular mechanism of collaboration between CR3 and Dectin-1 in macrophages. Our findings clearly showed that collaboration between CR3 and Dectin-1 in induction of TNF and IL-6 production was through synergistic activation of their downstream Syk-JNK-AP-1 signaling axis. In addition, while both CR3 and Dectin-1 were recruited and clustered on lipid raft microdomains upon encountering *H*. *capsulatum*, disruption of lipid raft hampered their collaboration in signaling activation and the subsequent cytokine response. Interestingly, CR3 and Dectin-1 cooperatively participated in host defense against disseminated histoplasmosis. Taken together, our results revealed the molecular mechanism underlying crosstalk between CR3 and Dectin-1 in enhancing cytokine response and demonstrated that they orchestrate adaptive antifungal immune response.

## Results

### CR3 and Dectin-1 collaborate to activate Syk in macrophage cytokine response

**Fig 1:**
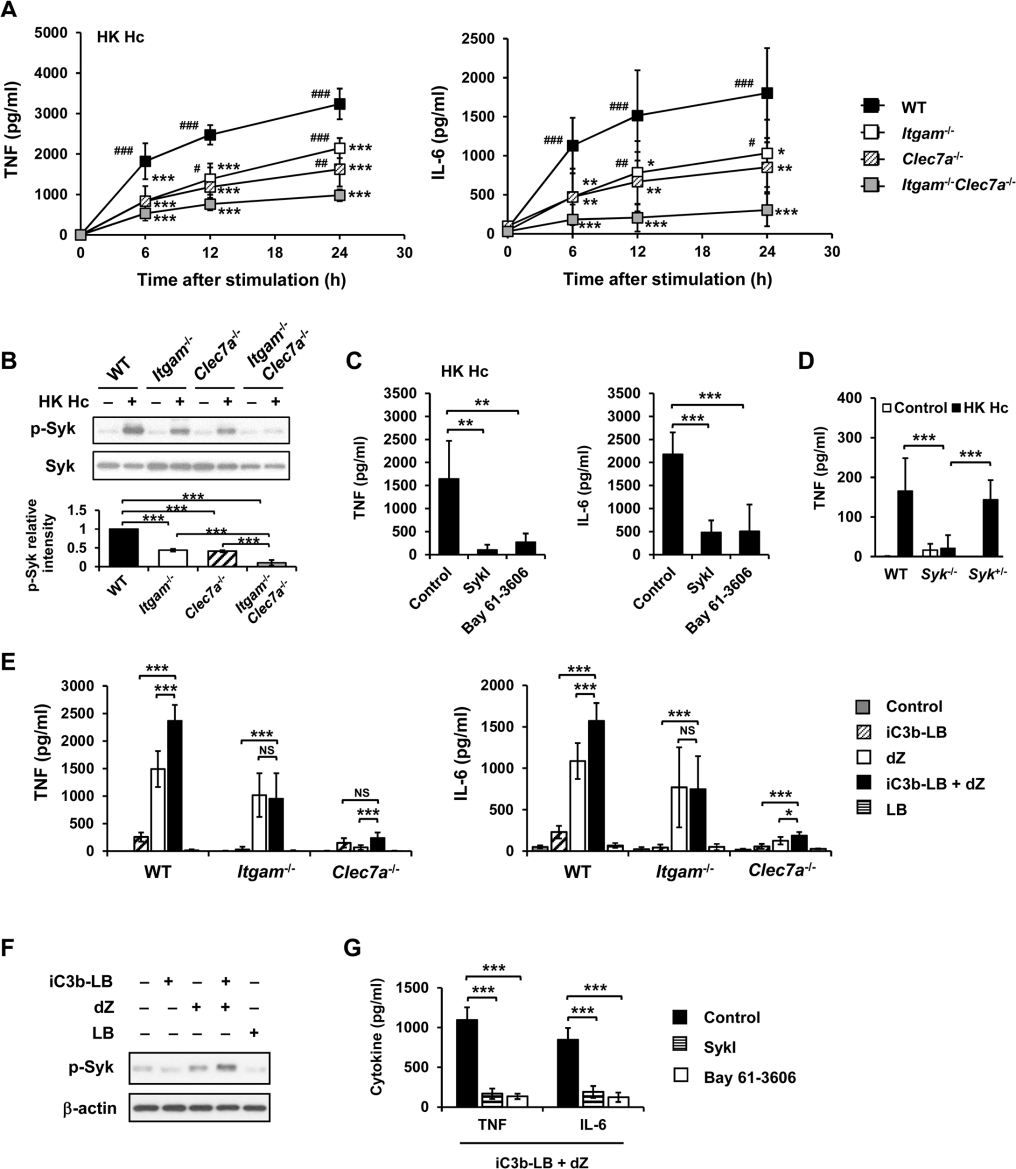
CR3 and Dectin-1 collaborate to intensify Syk activation and subsequent cytokine response in macrophage. (A and B) Macrophages from WT, *Itgam* ^-/-^, *Clec7a* ^-/-^ and *Itgam* ^-/-^ *Clec7a* ^-/-^ mice were stimulated with or without (0 h) heat-killed (HK) *H*. *capsulatum*. (A) Supernatants were collected at 6, 12, and 24 h after stimulation, and the concentrations of TNF and IL-6 in the supernatants were quantified by ELISA (n = 6 from 3 independent experiments). (B) Cell lysates were collected at 30 min after stimulation and analyzed by Western blotting using antibodies against p-Syk and Syk. The intensity of p-Syk was normalized against the corresponding Syk. Data shown in the lower panel are relative intensity of p-Syk (n = 3). (C and G) Macrophages from WT mice were treated with Syk inhibitors (SkyI, 10 μM; Bay 61-3606, 3 μM) starting at 1 h prior to stimulation with HK *H*. *capsulatum* (C) or with the combination of iC3b-coated beads (iC3b-LB) and depleted zymosan (dZ) (G). Culture supernatants were harvested 6 h later and analyzed for cytokine production (n = 5 from 2 independent experiments). (D) Fetal liver-derived macrophages (FLDMs) from *Syk* ^+/+^, *Syk* ^*-/-*^ and *Syk* ^*+/-*^ embryos were stimulated with HK *H*. *capsulatum* for 6 h. TNF levels in the culture supernatants were evaluated (n = 9 from 3 independent experiments). (E) Macrophages from WT, *Itgam* ^-/-^, and *Clec7a* ^-/-^ mice were stimulated with iC3b-LB, dZ, uncoated Latex beads (LB), or the combination of iC3b-LB and dZ. Culture supernatants were collected 6 h later and analyzed for cytokine production (n = 6 from 3 independent experiments). (F) Macrophages from WT mice were stimulated with or without iC3b-LB, dZ, LB, or the combination of iC3b-LB and dZ for 30 min. Cell lysates were subjected to Western blotting. Data are representative of 3 independent experiment with similar results. The bars represent the mean ± SD. In (A),* represent comparisons with WT and # with *Itgam* ^-/-^ *Clec7a* ^-/-^. * or # *p* ≦ 0.05, ** or ## *p* ≦ 0.01, *** or ### *p* ≦ 0.001. NS, not significant [one-way ANOVA with Tukey post-hoc test analysis (A, B, D and E); 2-tailed *t*-test (C and G)].

By use of blocking antibodies we previously showed that macrophage utilizes CR3 to phagocytose and both CR3 and Dectin-1 to mediate cytokine response to *H*. *capsulatum* [13]. Employing *Itgam* ^-/-^, *Clec7a* ^-/-^, and *Itgam* ^-/-^ *Clec7a* ^-/-^ macrophages here we investigated the mechanism of receptor crosstalk between CR3 and Dectin-1 (S1A Fig). While losing either or both CR3 and Dectin-1 did not affect the propagation of intracellular *H*. *capsulatum* (S2A Fig), TNF and IL-6 responses to either heat-killed or viable *H*. *capsulatum* were reduced in *Itgam* ^*-/-*^ and *Clec7a* ^*-/-*^ macrophages and the reduction was further enhanced in macrophages deficient in both receptors (Figs 1A and S3A, and also refer to S2B Fig). Blocking Dectin-1 in *Itgam* ^-/-^ macrophages or blocking CR3 in *Clec7a* ^-/-^ macrophages also revealed that signals from these two receptors had additive effect in TNF and IL-6 production (S4A Fig). It is worth noting that heat treatment did not change *H*. *capsulatum* recognition by macrophages.

Both CR3 and Dectin-1 are known to signal through activation of Syk kinase [21, 29]. We examined whether Syk is involved in the collaborative cytokine response induced by CR3 and Dectin-1. Stimulation with heat-killed or viable *H*. *capsulatum* activated Syk kinase and Syk activation was dampened in *Itgam* ^-/-^ and *Clec7a* ^-/-^ macrophages as well as in WT macrophages blocked by anti-CR3 or anti-Dectin-1 antibody (Figs 1B, S3B, S5A and S4B). The level of phosphorylated Syk was further reduced in *Itgam* ^-/-^ *Clec7a* ^-/-^ macrophages and in WT macrophages blocked by both anti-CR3 and anti-Dectin-1 antibodies (Figs 1B, S3B and S4B). These results suggest that signals from CR3 and Dectin-1 cooperatively activate Syk kinase. Treatment with Syk inhibitors significantly reduced *H*. *capsulatum*-induced TNF and IL-6 production and the reduction was not due to the cytotoxic effect of inhibitors (Figs 1C and S6). While Syk deficiency did not affect the expression of CR3 and Dectin-1, the deficiency almost completely abolished the production of TNF (Figs 1D and S1B). These results clearly demonstrate that CR3 and Dectin-1 act in concert in macrophage cytokine response to viable as well as heat-killed *H*. *capsulatum* by intensifying Syk activation.

To verify the nature of collaboration between receptors, specific particulate ligands for CR3 (iC3b-coated bead) and Dectin-1 (depleted zymosan) were used to stimulate cells. iC3b-coated beads, but not uncoated beads, and depleted zymosan additively induced TNF and IL-6 production in WT macrophages while *Itgam* ^-/-^ macrophages did not respond to stimulation by iC3b-coated beads neither did *Clec7a* ^-/-^ macrophages to depleted zymosan (Figs 1E and S7A). Co-stimulation of macrophages with iC3b-coated beads and depleted zymosan enhanced the level of phosphorylated Syk compared to stimulation by either ligand alone (Figs 1F and S7B) and treatment with Syk inhibitors abolished TNF and IL-6 response induced by these two ligands (Fig 1G). These data collectively reveal that Syk is the point where signals from CR3 and Dectin-1 converge to mediate collaborative cytokine response.

### CR3 and Dectin-1 clustering on lipid rafts facilitates their coordinated actions

**Fig 2:**
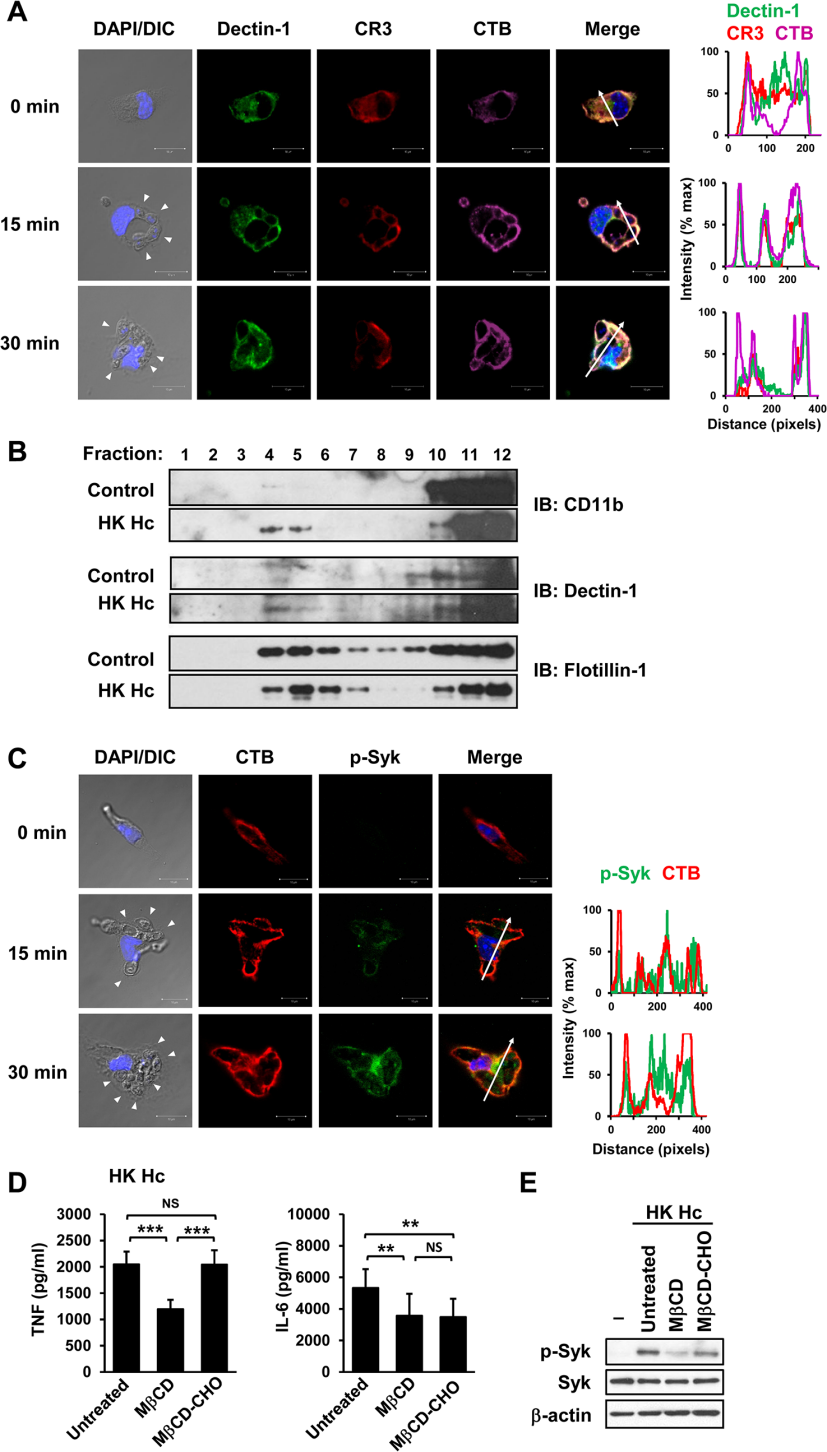
Clustering of CR3 and Dectin-1 on lipid rafts is required for their collaboration in cytokine production and signaling activation. (A and C) Macrophages were stimulated with or without (0 min) HK *H*. *capsulatum* for 15 or 30 min. Cells were fixed and stained for CR3 (red) and Dectin-1 (green) (A), or for p-Syk (green) (C). Cells were viewed under confocal microscope. Lipid raft was identified by staining with cholera toxin B (CTB) (violet). Nuclear compartment was stained by DAPI (blue). Arrowheads in the DIC/DAPI fields point to *H*. *capsulatum* yeasts. The intensity of different fluorochromes along the white arrow in the merged images is shown in the histogram on the right. (B) Macrophages from WT mice were stimulated with or without (control) HK *H*. *capsulatum* for 30 min. Cell lysates were subjected to sucrose gradient ultracentrifugation. The fractions were collected and subjected to Western blotting and probed with anti-CD11b, anti-Dectin-1 and anti-flotillin-1 antibodies. (D and E) Macrophages were untreated or treated with methyl-β-cyclodextrin (MβCD, 10 mM) for 30 min. To reconstitute cholesterol, MβCD-treated cells were cultured in medium containing water-soluble cholesterol (MβCD-CHO, 400 μg/ml) for 1 h. After washing, macrophages were stimulated with HK *H*. *capsulatum* for 6 h (D), or 30 min (E). The concentrations of TNF and IL-6 in culture supernatants were analyzed by ELISA. Mean ± SD are shown (n = 10 from 4 independent experiments) (D). Cell lysates were analyzed by Western blotting by using antibodies against p-Syk, Syk, and β-actin. Data are representative of 2 (A and C) and 3 (B and E) independent experiments with similar results. ** *p* ≦ 0.01, *** *p* ≦ 0.001. NS, not significant [one-way ANOVA with Tukey post-hoc analysis (D)].

It has been demonstrated that localization of Dectin-1 and CR3 to lipid raft microdomains is critical for signaling activation of each respective receptor [30, 31]. While both CR3 and Dectin-1 diffusely distributed in the cytosol and on cell membrane in unstimulated macrophages, they were recruited and colocalized on lipid raft microdomains at the interface between macrophage and *H*. *capsulatum* yeasts upon stimulation (Fig 2A). Isolation of lipid rafts by sucrose gradient confirmed that CR3 and Dectin-1 translocated to flotillin-1-enriched membrane microdomains (Fig 2B). Stimulating macrophages with *H*. *capsulatum* induced the phosphorylation of Syk which was associated with lipid raft microdomains (Fig 2C). It is noted that Syk was present in the lipid raft fractions on *H*. *capsulatum*-stimulated as well as on unstimulated cells, suggesting that rather than recruitment, *H*. *capsulatum* stimulation triggers phosphorylation of Syk that was already present on lipid rafts (S8A and S8B Fig). Disruption of lipid rafts by methyl-β-cyclodextrin (MβCD) significantly reduced TNF and IL-6 production and cholesterol replenishment rescued the ability to produce TNF but not IL-6 (Fig 2D). *H*. *capsulatum*-induced Syk phosphorylation was diminished in cells treated with MβCD and it was partially restored by cholesterol replenishment (Fig 2E). These results demonstrate that activated Syk is associated with lipid raft microdomains where CR3 and Dectin-1 cluster and the integrity of lipid rafts is important to macrophage cytokine response and signaling activation induced by *H*. *capsulatum*.

### Micro-Western Array analysis reveals the signaling pathways activated by *H*. *capsulatum*

**Fig 3:**
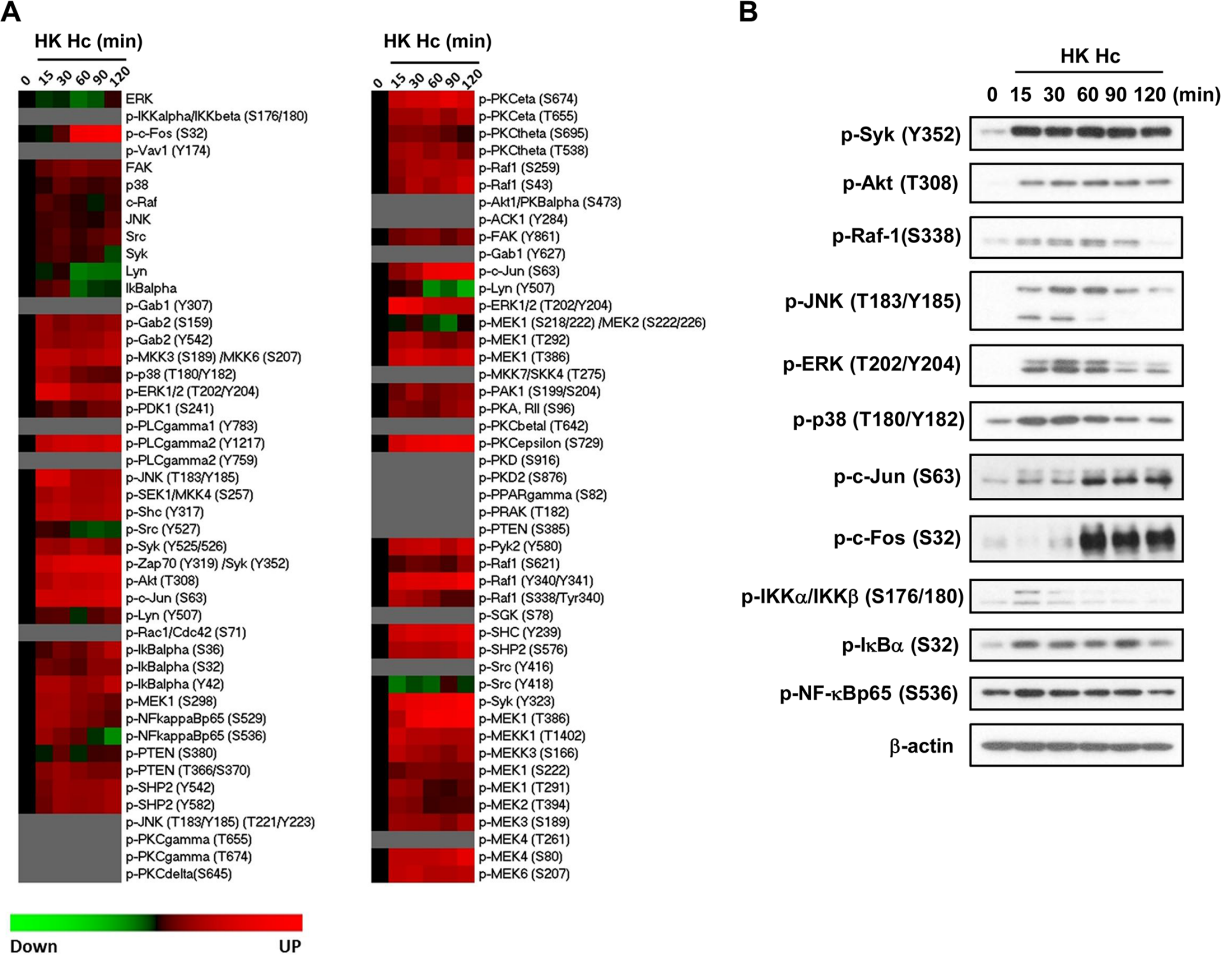
Micro-Western Array to screen for signaling molecules involved in *H*. *capsulatum*-induced macrophage response. Macrophages from WT mice were stimulated with or without (0 min) HK *H*. *capsulatum* for 15, 30, 60, 90, and 120 min. (A) Heat map chart shows MWA results. Protein abundance was normalized against the mean of β-actin and GAPDH. Black color indicates no change, while red and green indicate increase and decrease, respectively, of the levels of protein compared to unstimulated control. Proteins below the level of detection are in grey. (B) Cell lysates were subjected to Western blotting. Beta-actin was used as an internal control. Data shown are representative of 3 independent experiments with similar results.

We used Micro-Western Arrays (MWAs) by employing 92 antibodies to probe for phosphorylated (84 antibodies) and non-phosphorylated (8 antibodies) signaling proteins known to be involved in the pathways downstream of PRRs for phagocytosis and cytokine production to screen for signaling molecules activated by *H*. *capsulatum* (S9 Fig and S1 Table). The heat map shows that *H*. *capsulatum* induced phosphorylation of Syk, Raf-1, PLCγ2, PKC and molecules in the PI3K/Akt, NF-κB, and MAPK pathways at as early as 15 min and c-Jun and c-Fos (two components of AP-1) at 60 min after stimulation (Figs 3A and S9). Activated signaling molecules were validated by conventional Western blot analysis. Consistent with the MWA data, *H*. *capsulatum* stimulation caused the increase of phosphorylated Syk, Akt, Raf-1, JNK, ERK, p38, IKKα/IKKβ, IκBα and NF-κBp65 at 15 min, while phosphorylation of c-Jun and c-Fos occurred at 60 min after stimulation (Fig 3B). However, the amount of phosphorylated PLCγ2 and PKC isoforms (PKCε, PKCη, PKCθ) were below the limit of detection. Taken together, our data show that *H*. *capsulatum* stimulation leads to activation of Syk, Raf-1, AP-1, as well as molecules involved in the Akt/PI3K, NF-κB and MAPK pathways.

### JNK is involved in the crosstalk between CR3 and Dectin-1

**Fig 4:**
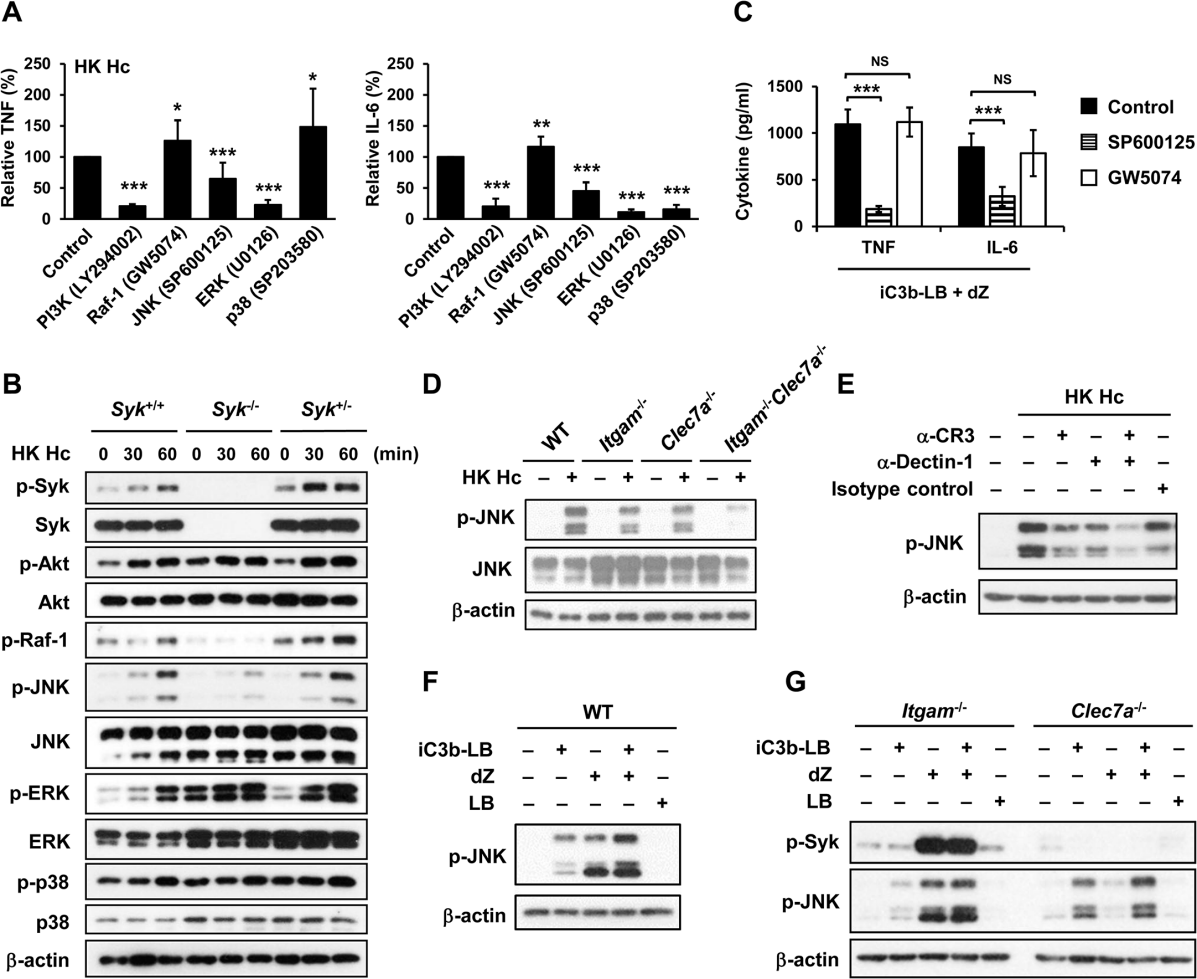
CR3 and Dectin-1 collaborate to enhance JNK activation. (A and C) Macrophages from WT mice were treated with vehicle (control) or different kinase inhibitors (PI3K inhibitor, LY294002 20 μM; Raf-1inhibitor, GW5074 1 μM; JNK inhibitor, SP600125 20 μM; ERK inhibitor, U0126 20 μM; p38 inhibitor, SP203580 20 μM) for 1 h prior to stimulation with HK *H*. *capsulatum* (A) or with the combination of iC3b-LB and dZ (C). Culture supernatants were collected 6 h later and the levels of TNF and IL-6 were quantified by ELISA. Mean ± SD of the relative (A) or absolute cytokine levels (C) of TNF and IL-6 are shown. [n = 5 from 3 independent experiments (A); n = 5 from 2 independent experiments (C)]. (B) FLDMs from *Syk* ^+/+^, *Syk* ^-/-^ and *Syk* ^*+/-*^ embryos were stimulated with or without (0 min) HK *H*. *capsulatum* for 30 or 60 min. Cell lysates were subjected to Western blotting. (D) Macrophages from WT, *Itgam* ^-/-^, *Clec7a* ^-/-^ and *Itgam* ^-/-^ *Clec7a* ^-/-^ mice were stimulated with or without (0 min) HK *H*. *capsulatum* for 30 min. Cell lysates were analyzed by Western blotting with the use of antibodies against p-JNK and JNK. (E) Macrophages from WT mice were treated with isotype control or blocking antibodies against CR3, Dectin-1, or both for 1 h before stimulation with HK *H*. *capsulatum*. Cell lysates were collected 30 min later and subjected to Western blotting. (F and G) Macrophages from WT (F) and *Itgam* ^-/-^ and *Clec7a* ^-/-^ (G) mice were stimulated with or without iC3b-LB, dZ, LB, or the combination of iC3b-LB and dZ for 30 min. Cell lysates were collected at 30 min after stimulation and subjected to Western blotting analysis. Data are representative of at least 3 independent experiments with similar results (B and D-G). * *p* ≦ 0.05, ** *p* ≦ 0.01, *** *p* ≦ 0.001. NS, not significant [2-tailed *t*-test (A and C)].

We used pharmacological kinase inhibitors to identify the signaling molecule(s) that participates in macrophage cytokine response to *H*. *capsulatum*. Treatment with PI3K, JNK and ERK inhibitors greatly diminished TNF and IL-6 production, yet inhibiting Raf-1 enhanced TNF and IL-6 responses (Fig 4A). Interestingly, p38 inhibitor had disparate effects on TNF and IL-6 production (Fig 4A). LDH assay showed that pharmacological inhibitors at the concentrations we used did not have cytotoxic effect on the cells (S6 Fig). Interestingly, Syk deficiency abrogated phosphorylation of Raf-1 and JNK but not that of Akt, ERK or p38 (Fig 4B) although inhibiting their activation diminished TNF and IL-6 production (Fig 4A). Results in Fig 4C show that inhibition of JNK activation in cells stimulated with iC3b-coated beads and depleted zymosan significantly reduced TNF and IL-6 production yet inhibition of Raf-1 did not affect the production of either cytokine. These results indicate that JNK, a signaling molecule downstream of Syk, plays an important role in the coordinated CR3 and Dectin-1cytokine response.

We next investigated the link between CR3 and Dectin-1 engagement and JNK activation. Results showed that JNK phosphorylation was reduced to about 50% of WT control in macrophages lacking either receptor across all time points after stimulation with heat-killed or viable *H*. *capsulatum* and JNK phosphorylation was almost completely abolished in *Itgam* ^*-/-*^ *Clec7a* ^*-/-*^ macrophages (Figs 4D, S3C and S5B). Similarly, blocking either receptor reduced JNK phosphorylation and blocking both receptors reduced JNK phosphorylation even further (Fig 4E). Stimulation of WT macrophages with both iC3b-coated beads and depleted zymosan increased the level of JNK phosphorylation compared to stimulation with either ligand alone (Fig 4F) and the additive effect of these two ligands was diminished in macrophages deficient in either receptor (Fig 4G). Taken together, our results show that engagement of CR3 and Dectin-1 separately induces JNK phosphorylation and engagement of both have an additive effect on JNK activation which is downstream of Syk.

### AP-1 mediates the collaborative cytokine response upon CR3 and Dectin-1 engagement

**Fig 5:**
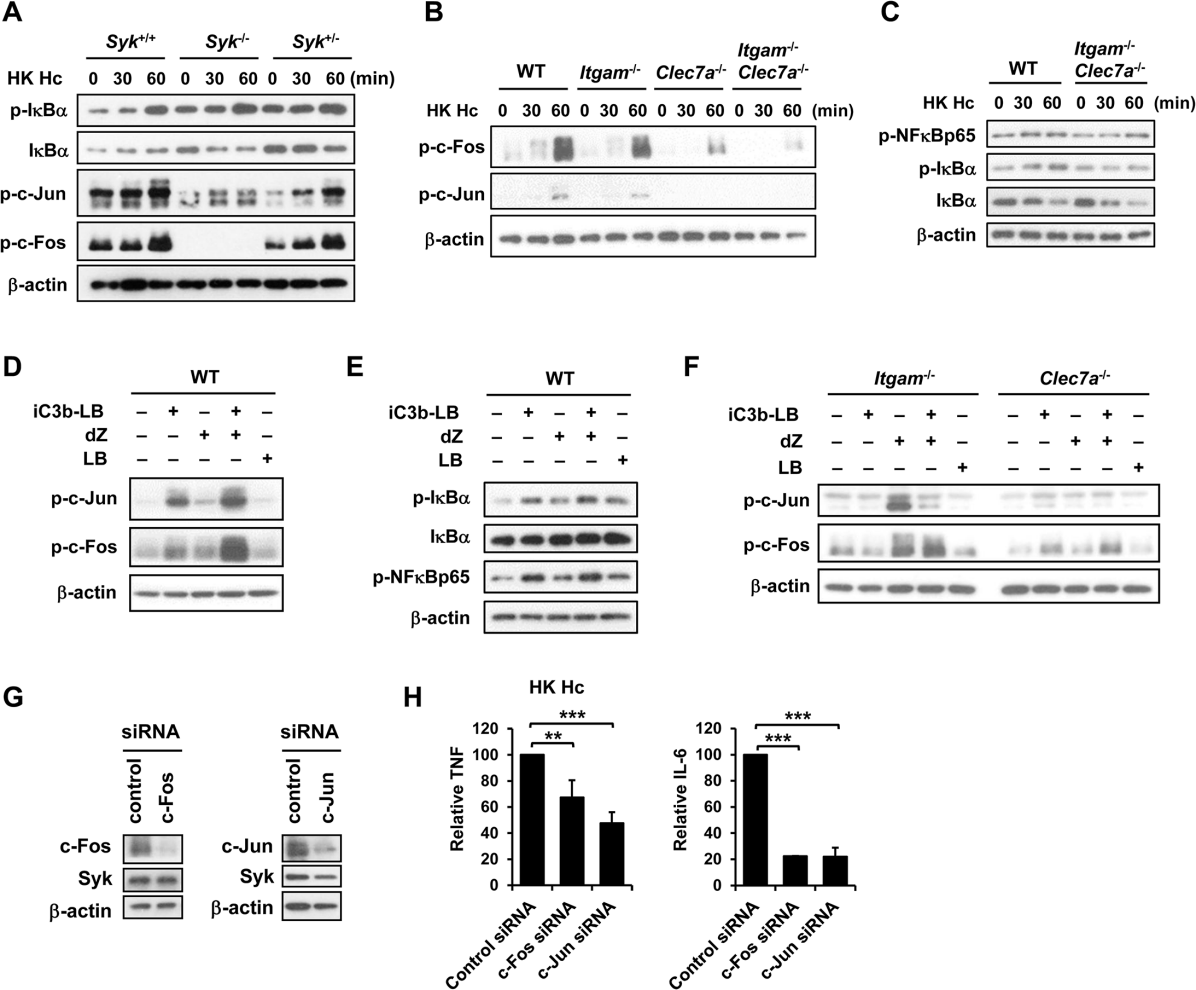
AP-1, but not NF-κB, mediates the collaborative cytokine response upon CR3 and Dectin-1 ligation. FLDMs from *Syk* ^+/+^, *Syk* ^-/-^ and *Syk* ^*+/-*^ embryos (A) and macrophages from WT, *Itgam* ^-/-^, *Clec7a* ^-/-^ and *Itgam* ^-/-^ *Clec7a* ^-/-^ mice (B and C) were stimulated with or without (0 min) HK *H*. *capsulatum* for 30 and 60 min. Cell lysates were analyzed by Western blotting. The blot shown in (A) is the same one shown in Fig 4B. Macrophages from WT mice (D and E), and *Itgam* ^-/-^ and *Clec7a* ^-/-^ mice (F) were stimulated with or without iC3b-LB, dZ, LB, or the combination of iC3b-LB and dZ for 30 min (E) or 60 min (D and F). Cell lysates were analyzed by Western blotting. Data are representative of at least 3 independent experiments (A-F). (G and H) Macrophages from WT mice were transfected with small interfering RNA (siRNA) against c-Fos or c-Jun, followed by stimulation with HK *H*. *capsulatum* 48 h later. Cell lysates and culture supernatants were collected at 6 h after stimulation. Silencing of c-Fos and c-Jun was confirmed by Western blotting (G). TNF and IL-6 levels in culture supernatants were quantified by ELISA and are presented as the mean ± SD of relative levels of TNF and IL-6 (n = 3 from 3 independent experiments) (H). ** *p* ≦ 0.01, *** *p* ≦ 0.001 [2-tailed *t*-test].

Results in Fig 3 show that stimulation of macrophages with *H*. *capsulatum* triggered the activation of both NF-κB and AP-1. To identify whether NF-κB or AP-1 mediates the collaboration between CR3 and Dectin-1 for cytokine production, we first clarified whether they were activated in *Syk* ^-/-^ macrophages. Fig 5A shows that *H*. *capsulatum*-induced c-Fos and c-Jun phosphorylation was greatly diminished in *Syk* ^-/-^ macrophages while IκBα phosphorylation and degradation was not affected, indicating that AP-1, but not NF-κB, is the transcription factor downstream of Syk. In addition, while IκBα and NF-κBp65 phosphorylation was not affected in single and double knockout macrophages, activation of c-Fos and c-Jun was dampened in *Itgam* ^-/-^ and *Clec7a* ^-/-^ macrophages and almost completely abolished in *Itgam* ^-/-^ *Clec7a* ^-/-^ macrophages (Figs 5B, 5C, S3D and S5C). Stimulation of WT macrophages with iC3b-coated beads and depleted zymosan together had an additive effect over stimulation with either one alone on c-Jun and c-Fos but not NF-κB activation (Fig 5D and 5E). The additive effect was abolished in single knockout macrophages (Fig 5F). When c-Jun and c-Fos were silenced by respective siRNA separately, TNF and IL-6 production was greatly reduced (Fig 5G and 5H). Together these data show that AP-1, but not NF-κB, is the transcription factor that mediates the collaborative cytokine response induced by CR3 and Dectin-1.

### CR3 and Dectin-1 concert in host defense against *H*. *capsulatum* infection

**Fig 6:**
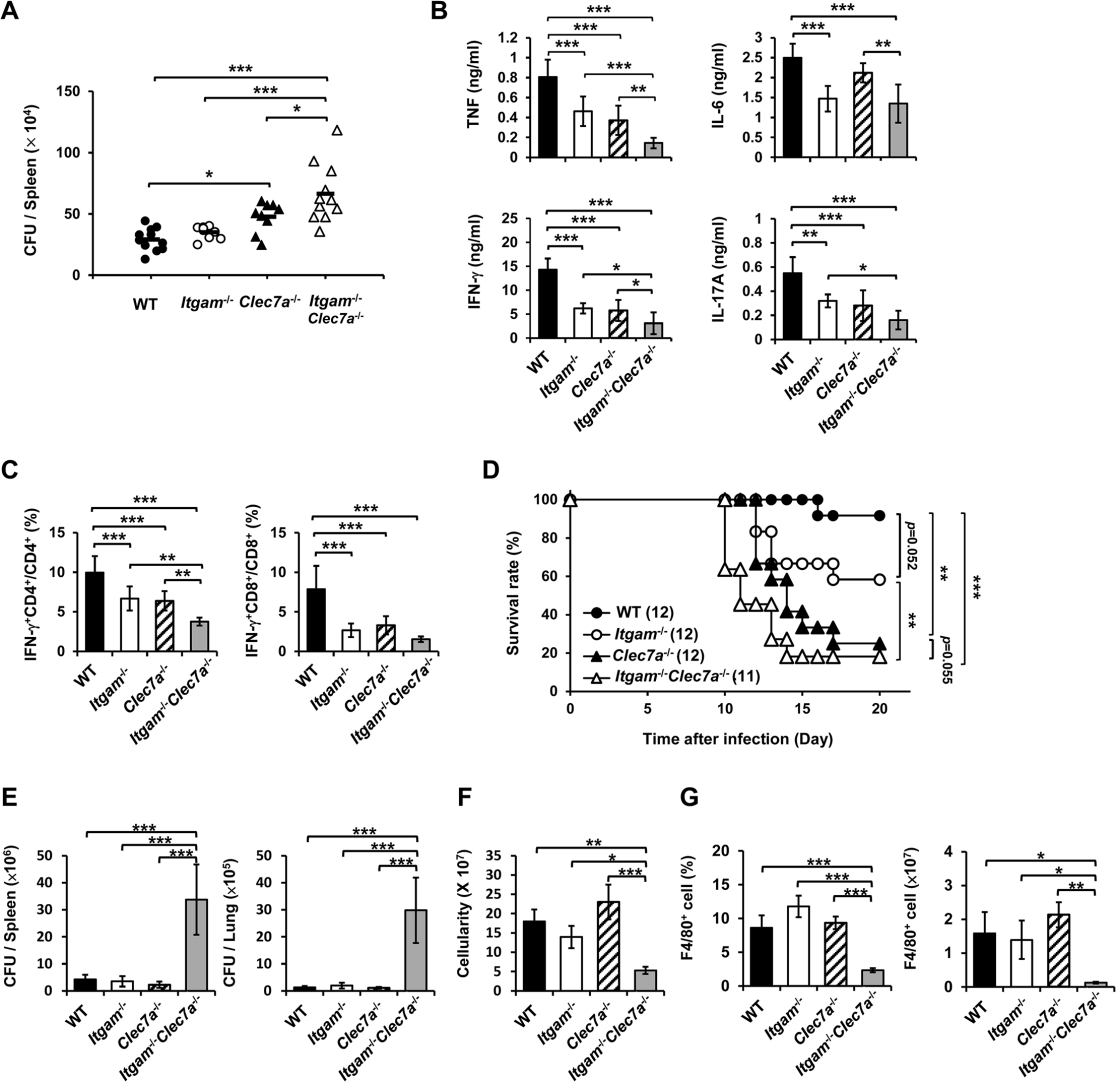
CR3 and Dectin-1 coordinately orchestrate host defense against *H*. *capsulatum* infection. (A-C) WT, *Itgam* ^-/-^, *Clec7a* ^-/-^, and *Itgam* ^-/-^ *Clec7a* ^-/-^ mice were infected with 2.5 × 10^5^ *H*. *capsulatum* intravenously. Infected mice were killed on day 7 after infection. (A) Spleen fungal burdens are shown as CFU per spleen. Data were pooled from 3 independent experiments. Each symbol represents one individual mouse. (B) Splenocytes were cultured for 48 h and the levels of TNF, IL-6, IL-17A and IFN-γ in the supernatants were quantified by ELISA. (C) Splenocytes were cultured for 24 h and stained for surface CD4 and CD8, and intracellular IFN-γ. Cells were analyzed by flow cytometry. Bar graphs show the percentage of IFN-γ ^+^ CD4^+^ and IFN-γ ^+^ CD8^+^ cells in total CD4^+^ and CD8^+^ T cell populations. Mean ± SD are shown (n = 6 from 3 independent experiments) (B and C). (D-G) WT, *Itgam* ^-/-^, *Clec7a* ^-/-^, and *Itgam* ^-/-^ *Clec7a* ^-/-^ mice were infected with 5 × 10^6^ *H*. *capsulatum* intravenously. Survival of infected mice is expressed as a Kaplan-Meier plot (D). Infected mice were killed on day 9 after infection (E-G). (E) The fungal burdens in the spleen and lung are shown as CFU per organ. (F) The total number of cells in the spleen was enumerated. (G) The percentage and the number of F4/80^+^ cells was analyzed by flow cytometry. Mean ± SD are shown (n = 3-4). * *p* ≦ 0.05, ** *p* ≦ 0.01, *** *p* ≦ 0.001 [one-way ANOVA with Duncan post-hoc analysis was used in data presented in (A-C and E-G); Generalized Wilcoxon test was used for data in (D)].

We employed disseminated histoplasmosis model, which is characterized by splenomegaly with large numbers of macrophages infiltrating the spleen, to investigate the contribution of CR3 and Dectin-1 in host defense against *H*. *capsulatum* infection [32]. WT, *Itgam* ^-/-^, *Clec7a* ^-/-^ and *Itgam* ^-/-^ *Clec7a* ^-/-^ mice were intravenously infected with a low sublethal dose of *H*. *capsulatum* (2.5 × 10^5^). While the fungal loads in *Itgam* ^-/-^ and *Clec7a* ^-/-^ single knockout mice were comparable to (*Itgam* ^-/-^) or slightly higher than (*Clec7a* ^-/-^) WT mice, that in double knockout mice were significantly higher than either of the single knockout mice on 7 days after infection (Fig 6A). Accompanying higher fungal loads, TNF, IFN-γ and IL-17 levels in splenocyte cultures of double knockout mice were lower than in either of the single knockout mice and IL-6 levels were lower than in *Clec7a* ^-/-^ mice (Fig 6B). Interestingly, the percentages of IFN-γ-producing CD4^+^ and CD8^+^ cells were significantly reduced in *Itgam* ^-/-^ and *Clec7a* ^-/-^ mice compared to WT mice, and they were reduced even further in double knockout mice (Fig 6C). Consistent with *in vivo* IFN-γ response, *Itgam* ^-/-^, *Clec7a* ^-/-^ and *Itgam* ^-/-^ *Clec7a* ^-/-^ dendritic cells stimulated with *H*. *capsulatum* produced significantly less IL-12p35 transcripts and IL-12p40 transcripts was reduced in *Itgam* ^-/-^ *Clec7a* ^-/-^ dendritic cells compared to WT cells (S10A and S10B Fig). These results together reveal that CR3 and Dectin-1 act in concert in instruction host adaptive immunity against *H*. *capsulatum*.

Infection with a higher dose of *H*. *capsulatum* (5 × 10^6^), *Itgam* ^-/-^ *Clec7a* ^-/-^ mice had significantly greater fungal burden, higher mortality and succumbed at an earlier time point (10 to14 days) compared to *Itgam* ^-/-^, *Clec7a* ^-/-^ or WT mice (Fig 6D and 6E). The number of total splenocytes in infected mice was significantly lower in *Itgam* ^-/-^ *Clec7a* ^-/-^ compared to either WT or single knockout mice (Fig 6F). Impressively, infection greatly reduced the proportion and number of F4/80^+^ cell population in *Itgam* ^-/-^ *Clec7a* ^-/-^ mice, which was significantly lower than in either of the single knockout mice and WT mice (Fig 6G and also refer to S11A and S11B Fig). These results suggest that macrophages in *Itgam* ^-/-^ *Clec7a* ^-/-^ mice fail to respond to *H*. *capsulatum* infection and/or rapidly undergo apoptosis. Macrophages being the major player in the interaction between the host and the fungal pathogen *H*. *capsulatum*, deficiency in innate receptors that recognize the pathogen for phagocytosis (CR3) and proinflammatory cytokine production (CR3 and Dectin-1) [13] greatly affects host defense against this pathogen. Furthermore, these two innate receptors CR3 and Dectin-1, collaboratively function not only in innate immune response but also orchestrate adaptive immune response against disseminated histoplasmosis.

## Discussion

Recognition of invading pathogens by PRRs triggers innate immune responses and shapes the adaptive immunity. Fungal cell wall is complex in its composition which varies between species, strains, and morphological forms. Innate immune cells use a unique set of PRRs to recognize and respond to a given fungus. Therefore, there is a lot of interest to dissect the molecular mechanism of coordination between heterogeneous PRRs. In the present study, we showed that CR3 and Dectin-1 collaborate in regulating macrophage pro-inflammatory cytokine response. Engagement of CR3 and Dectin-1 induces their clustering on lipid raft microdomains which function as a platform for downstream signaling. Utilizing MWA, genetic approach and pharmacological inhibitors, we demonstrated that receptor crosstalk between CR3 and Dectin-1 enhances the activation of their downstream Syk-JNK-AP-1 signaling pathway. Furthermore, our results showed that CR3 and Dectin-1 both participate and function cooperatively in host defense against *H*. *capsulatum* by facilitating adaptive antifungal immunity.

The pathogenic fungus *H*. *capsulatum* is assorted to chemotype I and II based on the absence and presence of α-(1,3)-glucan in the yeast cell wall [4]. *H*. *capsulatum* strain 505 we used in this study having its β-(1,3)-glucan readily exposed does not express α-(1,3)-glucan on the yeast cell surface which makes it likely to be assorted to chemotype I (S12A Fig). Rappleye *et al*. showed that *H*. *capsulatum* yeast strain G186A AGS (+) expressing α-(1,3)-glucan on the outer cell wall layer (S12B Fig) is not recognized by Dectin-1 [33]. The isogenic strain *ags1*-null mutant lacking α-(1,3)-glucan is recognized by Dectin-1-expressing cells and induces TNF production in phagocytic cells [33]. We showed in a previous study and here that strain 505 induces macrophage TNF and IL-6 production through both CR3 and Dectin-1, a phenomenon possibly unique to *H*. *capsulatum* that classified as chemotype I [13]. Interestingly, unlike what is reported in *C*. *albicans* [34], heat treatment does not alter β-glucan expression on *H*. *capsulatum* strain 505 (S12A and S12C Fig). We also discovered that Syk-JNK-AP-1 signaling and TNF and IL-6 production triggered by heat-killed *H*. *capsulatum* are comparable to that induced by viable organism.

In the case of PRR crosstalk, previous studies showed that Syk is required for the synergy between TLR and Dectin-1 and acts as the convergence point of Dectin-1 and Dectin-2 signaling, both of which situations lead to NF-κB activation [7, 9, 12, 35]. We demonstrated here that signaling crosstalk between CR3 and Dectin-1 through stimulation with *H*. *capsulatum* initiates at and converges on Syk. However, unlike receptor crosstalk between TLR and Dectin-1 and that between Dectin-1 and Dectin-2, activation of Syk through CR3 and Dectin-1 does not connect to NF-κB pathway. Instead, Syk leads to activation of JNK and AP-1. Consistent with *H*. *capsulatum* stimulation, co-stimulation with iC3b-coated beads and depleted zymosan enhances activation of Syk, JNK and AP-1, but not NF-κB. Our findings clearly define Syk-JNK-AP-1 axis as the signaling pathway downstream of the collaborative interaction between CR3 and Dectin-1.

CR3 is unique among the members of β2 integrin family that it contains two ligand binding sites, I domain and lectin-like domain. Single or dual ligation of I domain and lectin-like domain in CR3 triggers disparate signaling pathways and cellular responses [15, 17]. We have previously shown that CR3 recognizes *H*. *capsulatum* through both I domain and lectin-like domain within CD11b, demonstrating that *H*. *capsulatum* mediates dual CR3 ligation [13]. Here we observed that stimulation of macrophages with iC3b-coated beads (single CR3 ligation of I domain) does not induce Syk phosphorylation, while co-stimulation with iC3b-coated beads and depleted zymosan (dual CR3 ligation and Dectin-1 ligation) triggers Syk phosphorylation and the level of phosphorylation is greater than stimulation by depleted zymosan alone (single CR3 ligation of lectin-like domain and Dectin-1ligation). Interestingly, stimulating *Clec7a* ^-/-^ macrophages with both iC3b-coated beads and depleted zymosan only minimally activates Syk. The lack of inside-out signaling provided by Dectin-1 may account for the failure for iC3b-coated beads and depleted zymosan to activate CR3 [19]. Together, our results show that dual ligation of CR3 optimizes the signaling synergy induced by simultaneous engagement of CR3 and Dectin-1. An early study showed that *H*. *capsulatum* yeasts activate the alternative complement pathway which may lead to iC3b deposition [36]. Although CR3 can directly recognize and respond to *H*. *capsulatum*, whether complement opsonization would enhance the collaboration between CR3 and Dectin-1 or change PRR usage in macrophage interaction with *H*. *capsulatum* needs to be investigated.

PRR clustering in membrane lipid microdomains is crucial for host cells to optimize detection of fungal pathogens by formation of phagocytic synapse and serving as a platform for signaling synergy [31, 37, 38]. In this study, we show that the spatial nearness of CR3 and Dectin-1 and that both of them being mobilized to and associated with lipid rafts are important to achieve their collaboration upon engagement with *H*. *capsulatum*. However, little is known about how PRRs are recruited to lipid rafts and how signaling crosstalk is induced by heterogeneous PRRs. It has been reported that intracellular osteopontin (iOPN) is essential for clustering of heterologous PRRs, including Dectin-1, TLR2 and mannose receptor, that recognize *Pneumocystis* [38]. In addition, iOPN is involved in Dectin-1 and TLR2 downstream signaling by acting as a scaffold protein which associates with their respective downstream molecule Syk and IRAK1 [38]. It remains to be answered whether the steric assembly and the signaling synergy of CR3 and Dectin-1 induced by *H*. *capsulatum* is also mediated by iOPN, or by other adaptor molecule(s).

Macrophage plays multiple roles in host defense against *H*. *capsulatum*. Besides being a host cell, it also functions as cytokine/chemokine-producing cell and antigen donor cell, and it serves as effector cell when stimulated with IFN-γ, IL-17 and GM-CSF [39–42]. However, little is known about the signaling pathways downstream of functional receptors after macrophage encountering *H*. *capsulatum*. By MWA, this study is the first to uncover signaling pathways activated by *H*. *capsulatum* in macrophages. Our results reveal that PI3K, NF-κB, MAPK and AP-1 signaling pathways are activated by *H*. *capsulatum*. It is interesting to note that although PI3K, ERK and p38 activities modulate TNF and IL-6 production, they do not function downstream of Syk. In addition, inhibition of NF-κB does not affect *H*. *capsulatum*-induced TNF and IL-6 responses (S13 Fig). Thus, we postulate that other pathways may act in parallel with Syk-JNK-AP-1 pathway to regulate *H*. *capsulatum*-induced TNF and IL-6 response in macrophages.

We show that *H*. *capsulatum* induces Raf-1 activation in a Syk-dependent manner. Inhibition of Raf-1 increases *H*. *capsulatum*-induced TNF and IL-6 production. Our results define Raf-1 as a negative regulator in macrophage cytokine response to *H*. *capsulatum*. A previous study showed that Dectin-1 induces Raf-1 activation in a Syk-independent manner [26]. Activated Raf-1 antagonizes Syk-dependent non-canonical NF-κB activation by promoting inactive p65/RelB dimer formation [26]. Ligation of DC-SIGN activates Raf-1 which downregulates *Borrelia burgdorferi*- and TLR-induced TNF and IL-6 response by destabilizing their mRNAs and suppresses IL-12 response by impairing nucleosome remodeling at IL-12p35 promoter [43]. Together, these data suggest that activation of Raf-1 by ligation of PRRs negatively regulates and fine-tunes innate immune response. More studies are needed to demonstrate how Raf-1 negatively regulates *H*. *capsulatum*-induced TNF and IL-6 production.

There are only limited studies on the role of AP-1 in PRR crosstalk and in host defense against fungal infections. It has been shown that TLR2 and Dectin-1 cooperatively regulate zymosan-induced IL-10 production in DCs through an ERK-dependent, but AP-1-independent mechanism [44]. Other reports showed that Dectin-1 engagement in DCs triggers AP-1 activation, while curdlan stimulates a PLCγ2-dependent pathway, β-glucan on *Aspergillus fumigatus* activates a Syk-dependent pathway [22, 45, 46]. Our results demonstrate that CR3 acts in concert with Dectin-1 to activate both c-Jun and c-Fos. In addition, knocking down c-Jun and c-Fos in macrophages decreases *H*. *capsulatum*-induced TNF and IL-6 response, highlighting the role of AP-1 in host defense against fungal infections. Our MWA data show that PLCγ2 is activated by *H*. *capsulatum*. However, whether PLCγ2 is involved in CR3/Dectin-1-mediated AP-1 activation still remains to be determined. It is interesting to note that AP-1 activation is known to be associated with several disorders (ex. cancer and autoimmune diseases) by regulating genes involved in cell proliferation, angiogenesis and inflammation and inhibition of AP-1 activation is identified as a promising therapeutic strategy [47–49]. Our findings raise the possibility that administration of AP-1 inhibitor may increase susceptibility to fungal infections by suppressing proinflammatory cytokine production. Thus, AP-1inhibitor should be used with caution as a treatment modality for cancer and inflammatory disorder.

In addition to acting as a PRR to interact with pathogens, CR3 plays multiple roles in cellular processes including leukocyte extravasation, adhesion and chemotaxis [17]. This adds to the complexity of experimental design in addressing the role of CR3 *in vivo*. Intranasal or intraperitoneal infection of *Itgam* ^-/-^ mice with *S*. *pneumonia* results in infiltration of a large number of neutrophils to the infection sites [50, 51]. Neutrophil recruitment to the peritoneum is reduced in mice lacking CR3 after intraperitoneally challenge with *C*. *albicans*, yet accumulation of neutrophils in the kidney is comparable between WT and CR3-deficient mice intravenously infected with *C*. *albicans* [19, 52]. These studies demonstrated that abnormal neutrophil infiltration to the infection site should be considered as a factor influencing susceptibility in *in vivo* studies employing *Itgam* ^-/-^ mice. Indeed, we observed that unlike in WT mice, there is massive neutrophil infiltration to the lungs of *Itgam* ^-/-^ mice after intratracheal infection with *H*. *capsulatum*, a condition which may interfere the interaction between macrophages and the yeasts (S14A and S14B Fig). By contrast, the percentage and the number of neutrophils recruited to the spleen of *Itgam* ^-/-^ mice were commensurate with that in WT mice after intravenous inoculation of *H*. *capsulatum*. To focus on the roles of CR3 and Dectin-1 in macrophage interaction with *H*. *capsulatum*, we resorted to employ the disseminated histoplasmosis model by intravenous inoculation of the organism instead of pulmonary infection.

PRR signaling is known to act as a bridge that links innate and adaptive immunity. Signaling transduced by Dectin-1 can induce Th1 and Th17 responses [53] as well as priming cytotoxic T cells [54]. In addition, it has been reported that blockade of CR3 significantly reduces the Th1 and Th17 responses induced by *A*. *fumigatus* [55]. *H*. *capsulatum* infection triggers IFN-γ and IL-17 production by both CD4^+^ and CD8^+^ T cells, and both IFN-γ and IL-17 activate macrophages for inhibition of the replication of intracellular *H*. *capsulatum* [39, 41, 56, 57]. Mice deficient in IFN-γ or treated with neutralizing antibody against IFN-γ or IL-17 are more susceptible to *H*. *capsulatum*, showing the importance of IFN-γ and IL-17 in clearing this intracellular fungal pathogen [57–59]. However, whether and how PRRs participate in the development of *H*. *capsulatum*-induced IFN-γ and IL-17 response remains unclear. In this study, we show that both CR3 and Dectin-1 contribute to and function collaboratively in regulating TNF, IL-6, IL-17 and IFN-γ responses induced by *H*. *capsulatum*. TNF is a critical factor for the host to control *H*. *capsulatum*, and acts together with IFN-γ and IL-17 to provide protection [57, 60]. Although the role of IL-6 in histoplasomsis has not been well addressed, previous studies showed that the generation of *H*. *capsulatum*-induced IL-17 response is dependent on IL-6 and IL-6 deficiency leads to impairment of Th1 response in mice infected with *C*. *albicans* or *A*. *fumigatus* [57, 61, 62]. Our results also showed that, in addition to regulate macrophage TNF and IL-6 production, both CR3 and Dectin-1 are involved in IL-12 response in dendritic cells, suggesting the role of these receptors in regulating dendritic cells cannot be ignored. Moreover, our *in vitro* study showed that deficiency in either CR3 or Dectin-1 or both did not affect the intracellular growth of *H*. *capsulatum* in macrophages, strengthening the possibility that CR3 and Dectin-1 deficiency resulting in susceptibility to disseminated *H*. *capsulatum* infection is due to their roles in regulating IFN-γ and IL-17 responses. It is interesting to note that macrophages in the spleens of *Itgam* ^-/-^ *Clec7a* ^-/-^ mice were greatly diminished (almost exhausted) after infection with a lethal dose of *H*. *capsulatum*, presenting a picture that the macrophages are losing the battle to the fungal pathogen. While dendritic cells are major antigen-presenting cells and macrophage are the host, cytokine-producing cell and effector cell in infection by *H*. *capsulatum*, our study reveals that CR3 and Dectin-1 are of vital importance not only in their collaborative roles in macrophage cytokine production but also in instructing adaptive immune response against disseminated histoplasmosis.

In summary, we demonstrate for the first time the mechanism of receptor crosstalk between a member of the integrin family and CLR resulting in enhanced cytokine response. The collaboration between CR3 and Dectin-1 is through activation of Syk-JNK-AP-1 signaling pathway and dependent on formation of PRR clusters on lipid rafts. Our results also highlight the importance of CR3 and Dectin-1 in innate recognition that instructs antifungal adaptive immune response. Collectively, our findings provide a better understanding of the molecular mechanisms underlying the collaboration between CR3 and Dectin-1 and offer a valuable model for disentangling the intricacies of host-pathogen interactions.

## Materials and Methods

### Ethics statement

All animal experiments were undertaken in accordance with the Guidebook for the Care and Use of Laboratory Animals, 3^rd^ Ed., 2007, published by The Chinese-Taipei Society of Laboratory Animal Sciences, approved by the Institutional Animal Care and Use Committee (IACUC, Permit number: 20130231) of National Taiwan University College of Medicine.

### Fungus

*Histoplasma capsulatum* (Hc) strain 505 yeast cells were used in the whole study. Yeast cells were cultured at 37°C on brain heart infusion (BHI) agar supplemented with 1 mg/ml cysteine and 20 mg/ml glucose. Heat-killed yeast cells were prepared by treatment at 65°C for 2 h. To examine the surface expression of α-glucan and β-glucan, viable or heat-killed yeasts were fixed with 4% paraformaldehyde and stained with antibodies against α-(1,3)-glucan (Clone MOPC-104E) (Biolegend, San Diego, CA, USA) and β-(1,3)-glucan (Biosupplies, Parkville, Australia) followed by analysis with flow cytometry (BD FACSCanto II, BD Biosciences).

### Mice

*Itgam* ^-/-^ (Stock number: 003991) and wild-type C57BL/6 (Stock number: 000664) mice were originally purchased from the Jackson Laboratories (Bar Harbor, ME, USA) and *Clec7a* ^-/-^ mice were generated by Dr. Gordon D. Brown [63]. *Itgam* ^-/-^ *Clec7a* ^-/-^ mice were generated by crossing *Itgam* ^-/-^ and *Clec7a* ^-/-^ mice. Mice heterozygous for a deletion in the *Syk* locus (*Syk* ^+/-^) were obtained from Dr. Clifford Lowell (University of California, San Francisco, CA, USA) [64]. All strains used in this study were on C57BL/6 background. They were maintained and bred in the National Taiwan University College of Medicine Laboratory Animal Center (NTU CMLAC) or in the National Laboratory Animal Center (NLAC, Taiwan) under specific pathogen-free (SPF) conditions. *In vivo* infection experiments were performed following biosafety level 2 (BSL-2) guidelines.

### Cells

Peritoneal macrophages were collected by lavage from mice at 4 days after peritoneal injection of 1 ml of 3% thioglycollate medium (Sigma-Aldrich, St Louis, MO, USA). Macrophages deficient in Syk were derived from fetal liver cells obtained from *Syk* ^-/-^ mouse embryos (E13.5-E15.5). *Syk* ^-/-^ embryos were separated from *Syk* ^+/+^ and *Syk* ^+/-^ embryos by their exhibition of severe petechiae and confirmed by genotyping [65]. Single-cell suspensions from fetal liver tissues were cultured in 20% L929-cell conditioned medium for 7 days. Over 95% of the adherent cells were F4/80^+^ which were identified as fetal liver-derived macrophages (FLDMs).

### Reagents and antibodies

Syk inhibitors SykI and BAY 61-3606, PI3K inhibitor LY294002, ERK inhibitor U0126, JNK inhibitor SP600125, and p38 inhibitor SB203580 were purchased from Calbiochem-Merck (Darmstadt, Germany). Raf-1 inhibitor GW5074, methyl-β-cyclodextrin (MβCD), and water-soluble cholesterol were obtained from Sigma-Aldrich.

Antibodies against phospho (p)-Zap-70 (Tyr319)/Syk (Tyr352), p-Akt (Tyr308), p-c-Raf (Ser338), p-ERK1/2 (Thr202/Tyr204), p-JNK (Thr183/Tyr185), JNK, p-p38 (Thr180/Tyr182), p-IKKα/β (Ser176/180), p-NF-κBp65 (Ser536), p-IκBα (Ser32), IκBα, p-c-Jun (Ser63), c-Jun, p-c-Fos (Ser32), and c-Fos were purchased from Cell Signaling (Beverly, MA, USA). Anti-Syk, anti-β-actin, HRP-conjugated anti-rabbit IgG, and HRP-conjugated anti-mouse IgG antibodies were purchased from GeneTex Inc. (Irvine, CA, USA). Blocking antibodies against CR3 (clone 5C6) and Dectin-1 (clone 2A11) were purchased from Serotec (Oxford, UK). Antibodies for cell surface staining, anti-CD11b (clone M1/70), anti-CD18 (clone GAME-46), and APC-conjugated anti-F4/80 antibody were obtained from eBioscience (San Diego, CA, USA), and anti-Dectin-1 (clone 218820) was purchased from R&D Systems (Minneapolis, MN, USA).

### Stimulation with fungus and particulate ligands

Macrophages were stimulated with viable or heat-killed *H*. *capsulatum* yeasts (at a yeast-to-macrophage ratio of 20/1) or iC3b-caoted beads (at a bead-to-cell ration of 10/1) and 50 μg/ml depleted zymosan (InvivoGen, San Diego, CA, USA).The iC3b-coated beads were prepared as described previously [66]. Briefly, 2 × 10^8^ of 3 μm Latex beads (Sigma-Aldrich) were incubated with PBS containing 20 μg/ml human IgM (Sigma-Aldrich) at 37°C for 60 min. Beads were washed with PBS then resuspended in freshly isolated mouse serum (diluted 1:1 in PBS) and incubated at 37°C for another 20 min. Beads were washed with Hank's balanced salt solution then resupspended in RPMI 1640 medium supplemented with 10% heat-inactivated FBS. During the process, the classical pathway of complement cascade was activated, resulting in C3b deposition on the surface of beads where it was rapidly and completely converted to iC3b [67].

### Cytokine assays

Macrophages were stimulated with or without *H*. *capsulatum* or particulate ligands. Culture supernatants were collected after incubation at 37°C for different periods of time. The concentrations of TNF and IL-6 in the supernatants were quantified by enzyme-linked immunosorbent assay (ELISA) kit (eBioscience) following the manufacturer’s instructions.

### Western blotting

Cells were lysed by PhosphoSafe Extraction Reagent cell lysis buffer (Novagen, Madison, WI, USA). Whole cell lysates were subjected to electrophoresis at 10% sodium dodecyl sulfate polyacrylamide gel (SDS-PAGE), and transferred to PVDF membrane. The membrane was incubated in buffer containing primary antibody against molecule of interest, followed by HRP-conjugated secondary antibody. The blot was developed by chemiluminescence using ECL solution (Millipore, Billerica, MA, USA). For normalization, the intensity of blots was quantified by ImageJ software (NIH, Bethesda, MD, USA).

### Isolation of lipid raft fractions

Macrophages (3 × 10^7^) were lysed with 0.5% Brij in TNE buffer [25 mM Tris (pH 7.5), 150 mM NaCl, 5 mM EDTA, protease inhibitors, 1 mM Na_3_VO_4_, and 1 mM NaF] and let stand on ice for 1 h. Lysates were then mixed with equal volume of 80% sucrose in TNE buffer and overlaid with 30% and 5% sucrose in the same buffer. The gradients were centrifuged at 40,000 × g in a SW55Ti rotor (Beckman Coulter, Fullerton, CA, USA) at 4°C for 18 h. Twelve fractions were collected and the proteins in the fractions were subjected to electrophoresis at 10% SDS-PAGE and Western blot analysis by using antibodies against CD11b (GeneTex), Dectin-1 (Santa Cruz Biotechnology, Santa Cruz, CA, USA), Syk, p-Syk, and flotillin-1 (Cell Signaling).

### Immunofluorescence staining and confocal microscopy

Macrophages were allowed to adhere on cover slide overnight and stimulated with heat-killed *H*. *capsulatum* yeasts. After stimulation, cells were fixed with 3% paraformaldehyde followed by permeabilization with 0.5% Triton X-100. Cells were blocked with PBS containing 5% heat-inactivated FBS and stained with Alexa Fluor 647-conjugated cholera toxin B (Invitrogen, Carlsbad, CA, USA), anti-p-Syk (Cell Signaling), anti-Dectin-1 (Serotec), and/or PE-conjugated anti-CD11b (eBioscience) antibodies. Cells were then stained with secondary Alexa Fluor-conjugated secondary antibodies (Jackson ImmunoResearch, West Grove, PA, USA). Cell nuclei were stained with Hoechst 33258. The images were acquired with a Zeiss Axiovert 100TV confocal microscope (Carl Zeiss Inc., Jena, Germany) and analyzed by Zen software (Carl Zeiss Inc.) and ImageJ software (NIH).

### Micro-Western Arrays (MWAs)

Peritoneal macrophages were stimulated with or without heat-killed *H*. *capsulatum*. Cells were lysed at different time points, and Micro-Western Arrays (MWAs) were performed to measure protein expression as previously described [68]. Blots were analyzed by Odyssey analysis software (Li-Cor Biosciences, USA). Heat maps were created by using PermutMatrix software (LIRMM).

### siRNA transfection of macrophages

Peritoneal macrophages (1 × 10^6^) were transfected with 30 pmol of control siRNA or siRNAs targeting c-Fos or c-Jun (Santa Cruz Biotechnology) using the Amaxa Nucleofector kit for mouse macrophages (Lonza, Basel, Switzerland) with a Nucleofector II electroporation device (Lonza). After transfection, cells were gently resuspended in RPMI 1640 medium supplemented with 20% heat-inactivated FBS and plated in 12-well tissue culture plate. Adherent cells were collected for cytokine assay and Western blot analysis 48 h later.

### *H*. *capsulatum* infection, fungal burden and leukocyte populations in spleen

Mice were injected intravenously with low (2.5 × 10^5^) or high (5 × 10^6^) dose of *H*. *capsulatum* yeasts suspended in PRMI 1640 medium. For survival studies, mice were monitored for up to 30 days. For immunological studies, mice were killed on day 7 (low dose) or day 9 (high dose) after infection. To determine the fungal burden, spleens were weighted and homogenized in sterile RPMI 1640 medium. The homogenates were serially diluted and plated on glucose-pepton agar plates. Mycelial colonies were counted 10 days after incubation as described elsewhere [40]. To determine the percentage of leukocyte populations in spleen, splenocytes were stained for surface CD4, CD8, B220, F4/80, Ly6G and CD11c and analyzed by flow cytometry. All antibodies were purchased from eBioscience.

### Ex vivo cytokine production and intracellular cytokine staining

To study cytokine production by splenocytes, single cell suspensions were prepared from the spleen. Five million splenocytes were cultured in RPMI 1640 complete medium containing 400 pg/ml of rIL-2 for 48 h. The concentrations of TNF, IL-6, IL-17A and IFN-γ in the culture supernatants were quantified by ELISA. To analyze intracellular IFN-γ, 1 × 10^6^ splenocytes were cultured in RPMI 1640 complete medium for 24 h and monensin (2 μM, Sigma-Aldrich) was added at 6 h before harvest. Cells were stained with anti-CD4, anti-CD8 and anti-IFN-γ antibodies as described previously [40]. The percentage of IFN-γ-producing cells in the total CD4^+^ or CD8^+^ T cell populations was calculated by dividing the % of IFN-γ^+^CD4^+^ cells or IFN-γ^+^CD8^+^ by the % of CD4^+^ or CD8^+^ cells. All antibodies were purchased from eBioscience.

### Statistics

The comparisons between multiple groups were analyzed with one-way ANOVA followed by Tukey post-hoc test or by Duncan post-hoc analysis using SPSS 22.0 statistical software (IBM, Armonk, NY, USA). The differences between two groups were tested by two-tailed *t*-test. Generalized Wilcoxon test was used to analyze mouse survival. Differences were considered significant at a *P* value of < 0.05.

### Accession numbers

The accession numbers in the UniPortKB/SwissProt database of the proteins mentioned in this study are followed: CD11b, P05555; CD18, P11835; Dectin-1, Q6QLQ4; Syk, P43404; JNK, Q91Y86 (JNK1) and Q9WTU6 (JNK2); c-Fos, P01101; c-Jun, P05627; Raf-1, Q99N57; PLCγ2, Q8CIH5; Akt, P31750; ERK, Q63844 (ERK1) and P63085 (ERK2); p38, P47811; IKKα, Q60680; IKKβ, O88351; IκBα, Q9Z1E3; NF-κBp65, Q04207; PKCε, P16054; PKCη, P23298; PKCθ, Q02111; β-actin, P60710; GAPDH, P16858; flotillin-1, O08917; TNF, P06804; IL-6, P08505; IL-12p35, P43431; IL-12p40, P43432; IL-17A, Q62386; IFN-γ, P01580.

## Supporting Information

S1 Fig Surface expression of CR3 and Dectin-1 in macrophages. (A) CR3 and Dectin-1 deficiency does not affect the surface expression of Dectin-1 and CR3, respectively. Macrophages from WT, *Itgam* ^-/-^, *Clec7a* ^-/-^, and *Itgam* ^-/-^ *Clec7a* ^-/-^ mice were stained for surface expression of Dectin-1, CD11b, CD18 and analyzed by flow cytometry. (B) Fetal liver-derived macrophages from *Syk* ^+/+^, *Syk* ^*-/*-^ and *Syk* ^*+/-*^ embryos were stained for surface expression of CD11b and Dectin-1 and analyzed by flow cytometry. Histogram shows the fluorescence intensity of each receptor gated on F4/80^+^ cells.
(TIF)

S2 Fig The effect of CR3 and Dectin-1 deficiency in macrophage phagocytosis and support of intracellular *H*. *capsulatum* replication. (A) Lacking either or both CR3 and Dctin-1 in macrophages does not affect the replication of intracellular *H*. *capsulatum*. Macrophages from WT, *Itgam* ^-/-^, *Clec7a* ^-/-^ or *Itgam* ^-/-^ *Clec7a* ^-/-^ mice were cultured with live *H*. *capsulatum* for 1 h followed by wash to rid unenglufed yeasts. Cells were lysed immediately (0 h) or after 18 h of incubation, and the number of yeast cells was counted. Replication time (h) = incubation interval/number of divisions; and number of divisions =log 2 (N_t_/N_0_), where N_t_ is the mean number of yeasts/infected macrophage at the end of incubation (18 h), and N_0_ is the mean number of yeasts/infected macrophage at time zero (0 h). (B) CR3, but not Dectin-1, is involved in macrophage phagocytosis of *H*. *capsulatum*. Macrophages from WT, *Itgam* ^-/-^, *Clec7a* ^-/-^ or *Itgam* ^-/-^ *Clec7a* ^-/-^ mice were allowed to phagocytose FITC-labeled HK *H*. *capsultaum* for 1 h. Percentages of cells taking up *H*. *capsulatum* were analyzed by flow cytometey. Mean ± SD are shown (n = 3). * *p* ≦ 0.05. NS, not significant [one-way ANOVA with Tukey post-hoc analysis].
(TIF)

S3 Fig Viable *H*. *capsulatum* activates macrophage Syk-JNK-AP-1 pathway and cytokine response through CR3 and Dectin-1. Macrophages from WT, *Itgam* ^-/-^, *Clec7a* ^-/-^ or *Itgam* ^-/-^ *Clec7a* ^-/-^ mice were stimulated with or without live *H*. *capsulatum*. (A) Culture supernatant were collected at 6 h after stimulation and evaluated for TNF and IL-6 production. Data shown are the mean ± SD (n = 3-4). (B-D) Cell lysate were collected at 30 min (B and C) or 60 min (D) after stimulation and analyzed by Western blotting. * *p* ≦ 0.05, ** *p* ≦ 0.01, *** *p* ≦ 0.001 [one-way ANOVA with Tukey post-hoc test analysis (A)].
(TIF)

S4 Fig Antibody blockade of CR3 and Dectin-1 reveals their collaboration in *H*. *capsulatum*-induced cytokine response and Syk activation. (A) Macrophages from *Itgam* ^-/-^ and *Clec7a* ^-/-^ mice were treated with anti-CR3 and anti-Dectin-1 blocking antibodies, reciprocally, or by isotype control at 5 μg/ml for 1 h prior to stimulation with HK *H*. *capsulatum* for another 6 h. The concentrations of TNF and IL-6 in culture supernatants were quantified by ELISA. Data shown are the mean ± SD of relative TNF and IL-6 (n = 5). (B) Macrophages from WT mice were treated with isotype control or blocking antibodies against CR3, Dectin-1, or both for 1 h before stimulation with HK *H*. *capsulatum*. Cell lysates were collected 30 min later and analyzed by Western blotting for Syk activation. *** *p* ≦ 0.001 [2-tailed *t*-test].
(TIF)

S5 Fig Syk, JNK, and AP-1 activation is reduced in macrophages deficient in CR3 or Dectin-1 after stimulation with *H*. *capsulatum*. Macrophages from WT, *Itgam* ^-/-^, *Clec7a* ^-/-^ mice were stimulated with or without (0 min) HK *H*. *capsulatum* for 15, 30 and 60 min. Cell lysates were analyzed by Western blotting for activated Syk (A), JNK (B), AP-1 and NF-κB (C). The intensity of p-Syk (A), p-JNK (B), p-IκBα, IκBα and p-NF-κBp65 at 30 min and p-c-Jun and p-c-Fos at 60 min (C) after stimulation was normalized against the corresponding internal controls. Data shown in the right panel of (A-C) are the mean ± SD of relative intensity (n = 5). ** *p* ≦ 0.01, *** *p* ≦ 0.001 [2-tailed *t*-test].
(TIF)

S6 Fig Treatment with kinase inhibitors does not cause cell death. LDH release by macrophages cultured in medium containing vehicle (control) or indicated kinase inhibitors for 7 h. Data shown are the mean ± SD of the percentage of LDH release (n = 3).
(TIF)

S7 Fig Co-stimulation with uncoated Latex beads and depleted zymosan does not have additive effect on either cytokine response or Syk activation. Macrophages from WT mice were stimulated with or without uncoated Latex beads (LB), depleted zymosan (dZ), or their combination for 6 h (A) or 30 min (B). (A) The concentrations of TNF and IL-6 in culture supernatants were quantified by ELISA. Mean ± SD are shown (n = 4). (B) Cell lysates were collected and analyzed by Western blotting for Syk activation. NS, not significant [2-tailed *t*-test].
(TIF)

S8 Fig Expression of Syk in lipid rafts. Macrophages were stimulated with or without HK *H*. *capsulatum* for 30 min. (A) Cell lysates were subjected to sucrose gradient ultracentrifugation. The presence of Syk in each fraction was analyzed by Western blotting. The blot probed with anti-flotillin-1 antibody was used to identify lipid raft fractions. (B) Bar graph shows the relative ratio of Syk to flotillin-1. That in fraction 4 of unstimulated cells was set as 1. Data presented are the mean ± SD (n = 3).
(TIF)

S9 Fig Micro-Western Array screening for signaling protein activation in macrophages stimulated with *H*. *capsulatum*. The signaling molecules in macrophages stimulated with or without HK *H*. *capsulatum* were screened by Micro-Western Array (MWA) to measure the changes in abundance of indicated proteins. The six samples printed in each well from left to right are macrophages unstimulated (0 min), and stimulated with HK *H*. *capsulatum* at a yeast-to-cell ratio of 20/1 for 15, 30, 60, 90, and 120 min. The red and green signals represent samples probed with secondary anti-rabbit and anti-mouse antibodies, respectively. The fluorochrome intensities were analyzed by Odyssey analysis software. S1 Table lists the antibodies used for blotting in each well of the 96-well array.
(TIF)

S10 Fig CR3 and Dectin-1 are involved in dendritic cell IL-12 response to *H*. *capsulatum*. BMDCs from WT, *Itgam* ^-/-^, *Clec7a* ^-/-^ and *Itgam* ^-/-^ *Clec7a* ^-/-^ mice were stimulated with or without live *H*. *capsulatum* (MOI = 2) for 6 h. The expression levels of IL-12p35 (A) and IL-12p40 (B) mRNA were analyzed by real-time qPCR. Data shown are the mean ± SD of relative transcript normalized against GAPDH (n = 3). * *p* ≦ 0.05, *** *p* ≦ 0.001 [one-way ANOVA with Tukey post-hoc analysis].
(TIF)

S11 Fig Percentage and number of leukocyte populations in spleens from *H*. *capsulatum*-infected mice. WT, *Itgam* ^-/-^, *Clec7a* ^-/-^, and *Itgam* ^-/-^ *Clec7a* ^-/-^ mice were infected with 5 × 10^6^ *H*. *capsulatum* intravenously. Infected mice were killed on day 9 after infection. The percentage (A) and number (B) of CD4^+^, CD8^+^, B220^+^, CD11c^+^ and Ly6G^+^ cells in the spleen was analyzed by flow cytometry. Mean ± SD are shown (n = 3-4). * *p* ≦ 0.05, ** *p* ≦ 0.01, *** *p* ≦ 0.001 [one-way ANOVA with Duncan post-hoc analysis].
(TIF)

S12 Fig Expression of α-(1,3)-glucan and β-(1,3)-glucan on *H*. *capsulatum* and C. albicans. (A) *H*. *capsulatum* strain 505 has β-glucan exposed and lacks α-glucan expression on the yeast cell wall. Live or HK *H*. *capsulatum* was stained with anti-α-glucan or anti-β-glucan antibody in the presence or absence of laminarin and analyzed by flow cytometry. (B) *H*. *capsulatum* strain G186A expressing α-glucan masks β-glucan on the yeast cell wall. Viable wild-type or *ags1*-null mutant *H*. *capsulatum* strain G186A were stained for surface expression of α-(1,3)-glucan and β-(1,3)-glucan and analyzed by flow cytometry. (C) Heat treatment exposes β-glucan on the surface of *C*. *albicans*. Viable or HK *C*. *albicans* strain SC5314 were stained for surface β-(1,3)-glucan and analyzed by flow cytometry.
(TIF)

S13 Fig Inhibition of NF-κB does not affect *H*. *capsulatum*-induced TNF and IL-6 responses. Macrophages from WT mice were treated with vehicle (control) or NF-κB inhibitor (SN50, 50 μg/ml) for 1 h prior to stimulation with HK *H*. *capsulatum*. Culture supernatants were collected 6 h later and the levels of TNF and IL-6 were quantified by ELISA. Mean ± SD are shown (n = 5). NS, not significant [2-tailed *t*-test].
(TIF)

S14 Fig Higher neutrophil infiltration in lungs of *Itgam* ^-/-^ mice with pulmonary *H*. *capsulatum* infection. WT and *Itgam* ^-/-^ mice were infected with 2.5 × 10^5^ *H*. *capsulatum* intratracheally. Mice were killed on day 7 post-infection. Leukocytes were isolated from lung homogenates and analyzed by flow cytometry after staining with surface CD45 and Ly6G. (A) Percentage of CD45^+^Ly6G^+^ cells in CD45^+^ population. (B) Number of CD45^+^Ly6G^+^ cells in the lungs. Mean ± SD are shown (n =3). * *p* ≦ 0.05, ** *p* ≦ 0.01 [2-tailed *t*-test].
(TIF)

S1 Table Antibodies used in Micro-Western Array. (DOCX)
