# Supplementary figures and images for: CR3 and Dectin-1 Collaborate in Macrophage Cytokine Response through Association on Lipid Rafts and Activation of Syk-JNK-AP-1 Pathway
Source: PLoS Pathog. 2015 Jul 1;11(7):e1004985. doi: 10.1371/journal.ppat.1004985 (PMC4488469; doi:10.1371/journal.ppat.1004985)

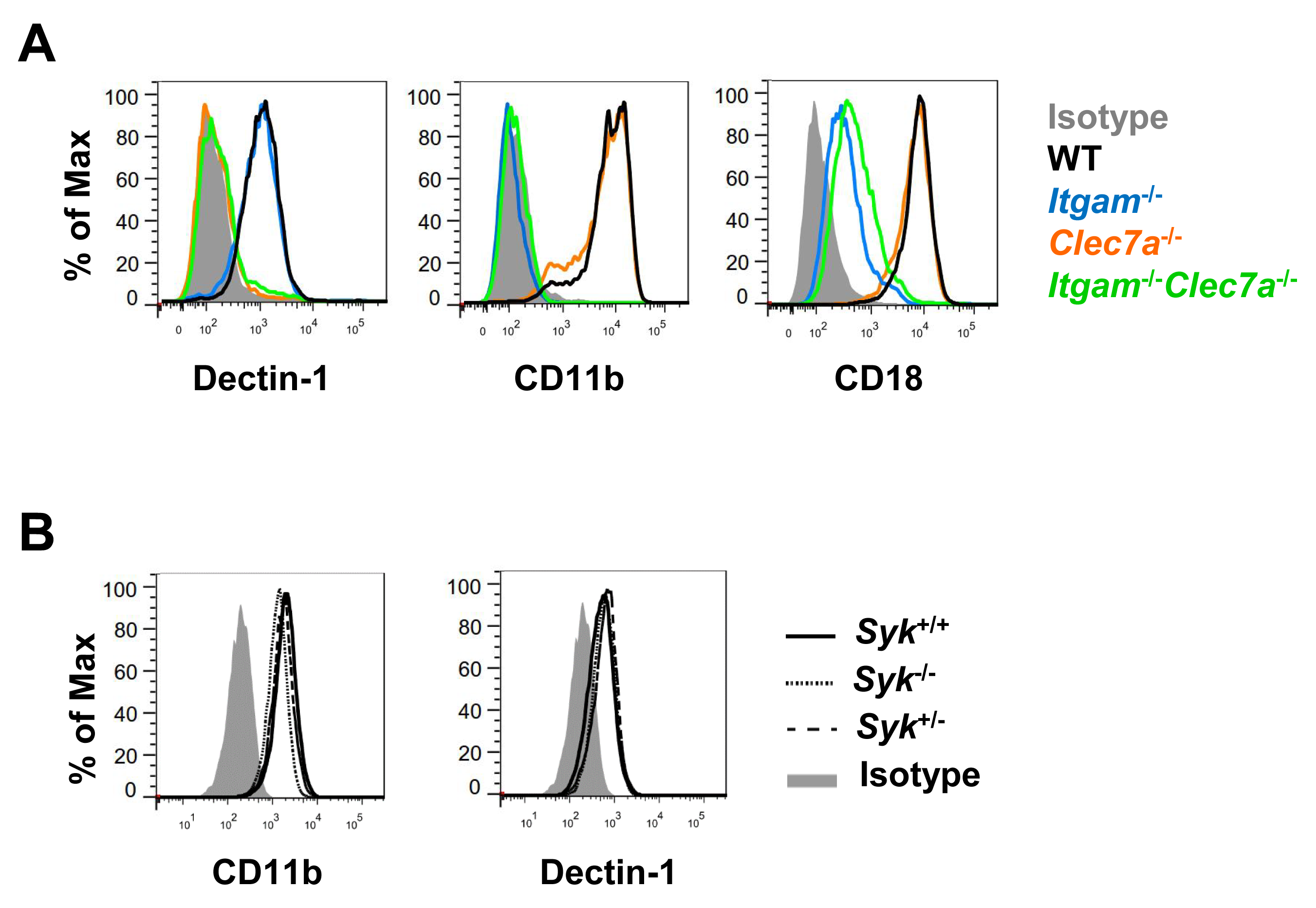

Supplement: S1 Fig — (A) CR3 and Dectin-1 deficiency does not affect the surface expression of Dectin-1 and CR3, respectively. Macrophages from WT, Itgam -/-, Clec7a -/-, and Itgam -/- Clec7a -/- mice were stained for surface expression of Dectin-1, CD11b, CD18 and analyzed by flow cytometry. (B) Fetal liver-derived macrophages from Syk +/+, Syk -/- and Syk +/- embryos were stained for surface expression of CD11b and Dectin-1 and analyzed by flow cytometry. Histogram shows the fluorescence intensity of each receptor gated on F4/80+ cells. (TIF) [file ppat.1004985.s001.tif]

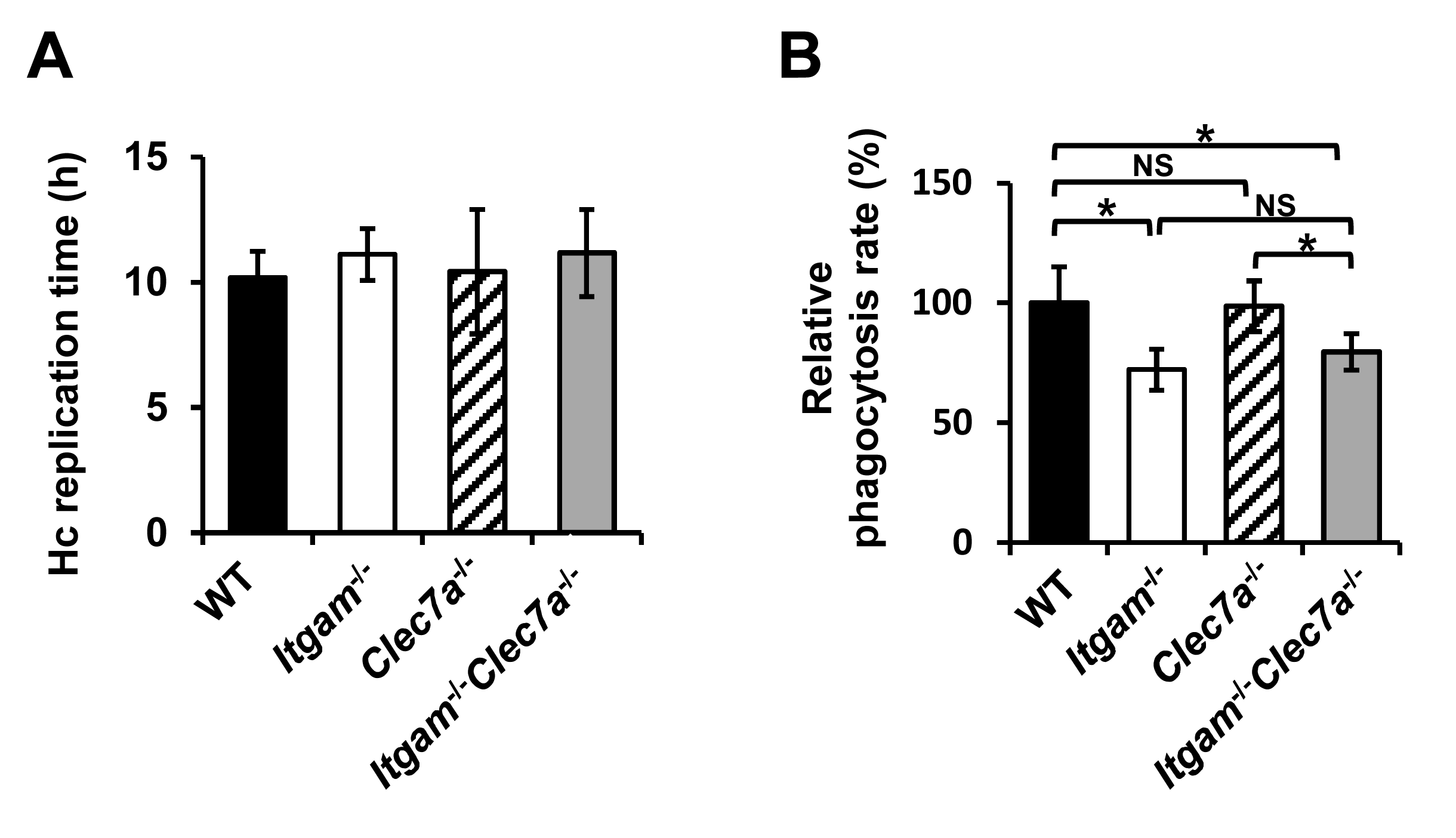

Supplement: S2 Fig — (A) Lacking either or both CR3 and Dctin-1 in macrophages does not affect the replication of intracellular H. capsulatum. Macrophages from WT, Itgam -/-, Clec7a -/- or Itgam -/- Clec7a -/- mice were cultured with live H. capsulatum for 1 h followed by wash to rid unenglufed yeasts. Cells were lysed immediately (0 h) or after 18 h of incubation, and the number of yeast cells was counted. Replication time (h) = incubation interval/number of divisions; and number of divisions =log 2 (Nt/N0), where Nt is the mean number of yeasts/infected macrophage at the end of incubation (18 h), and N0 is the mean number of yeasts/infected macrophage at time zero (0 h). (B) CR3, but not Dectin-1, is involved in macrophage phagocytosis of H. capsulatum. Macrophages from WT, Itgam -/-, Clec7a -/- or Itgam -/- Clec7a -/- mice were allowed to phagocytose FITC-labeled HK H. capsultaum for 1 h. Percentages of cells taking up H. capsulatum were analyzed by flow cytometey. Mean ± SD are shown (n = 3). * p ≦ 0.05. NS, not significant [one-way ANOVA with Tukey post-hoc analysis]. (TIF) [file ppat.1004985.s002.tif]

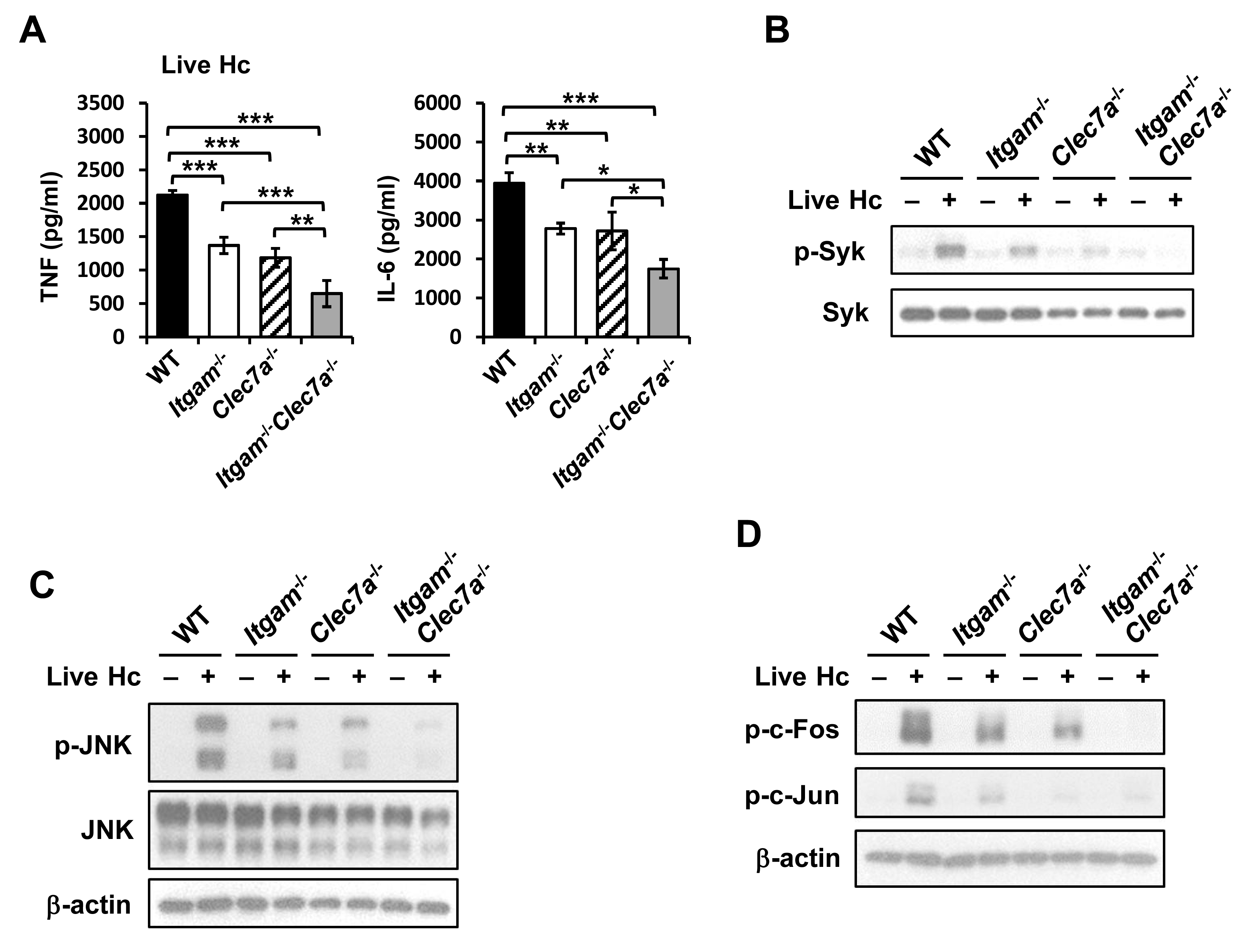

Supplement: S3 Fig — Macrophages from WT, Itgam -/-, Clec7a -/- or Itgam -/- Clec7a -/- mice were stimulated with or without live H. capsulatum. (A) Culture supernatant were collected at 6 h after stimulation and evaluated for TNF and IL-6 production. Data shown are the mean ± SD (n = 3-4). (B-D) Cell lysate were collected at 30 min (B and C) or 60 min (D) after stimulation and analyzed by Western blotting. * p ≦ 0.05, ** p ≦ 0.01, *** p ≦ 0.001 [one-way ANOVA with Tukey post-hoc test analysis (A)]. (TIF) [file ppat.1004985.s003.tif]

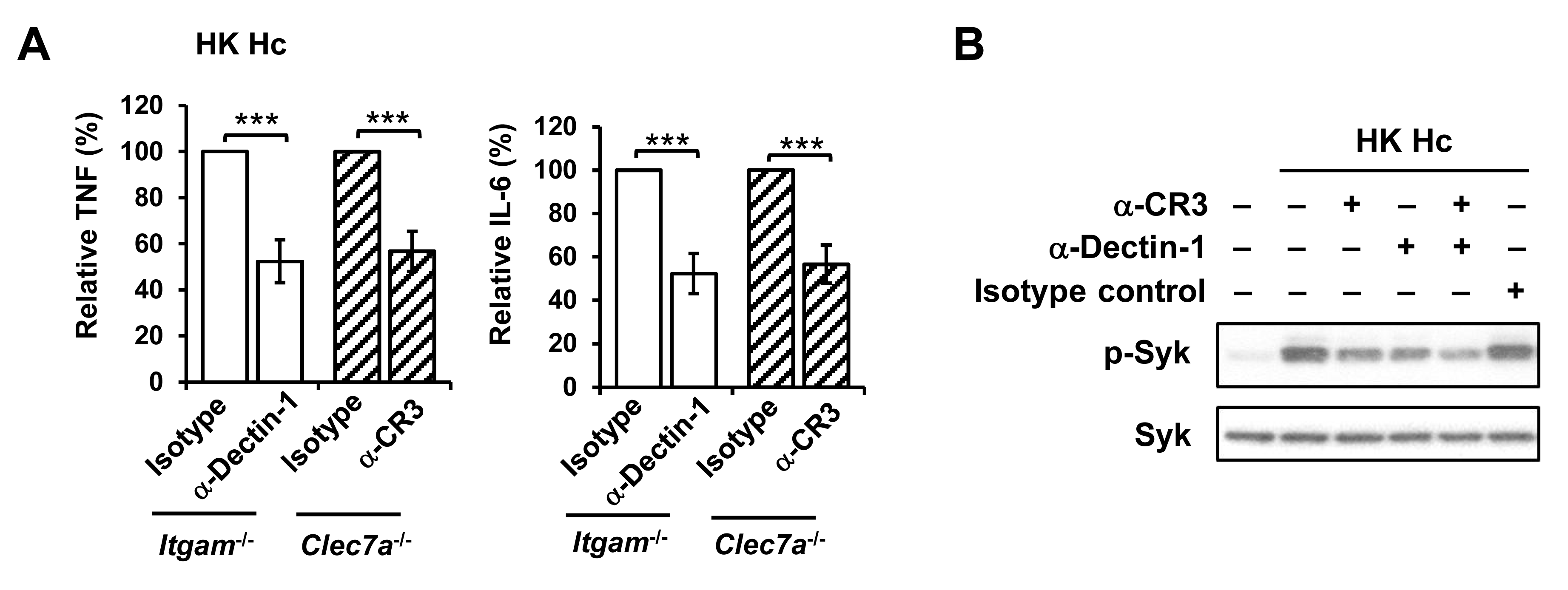

Supplement: S4 Fig — (A) Macrophages from Itgam -/- and Clec7a -/- mice were treated with anti-CR3 and anti-Dectin-1 blocking antibodies, reciprocally, or by isotype control at 5 μg/ml for 1 h prior to stimulation with HK H. capsulatum for another 6 h. The concentrations of TNF and IL-6 in culture supernatants were quantified by ELISA. Data shown are the mean ± SD of relative TNF and IL-6 (n = 5). (B) Macrophages from WT mice were treated with isotype control or blocking antibodies against CR3, Dectin-1, or both for 1 h before stimulation with HK H. capsulatum. Cell lysates were collected 30 min later and analyzed by Western blotting for Syk activation. *** p ≦ 0.001 [2-tailed t-test]. (TIF) [file ppat.1004985.s004.tif]

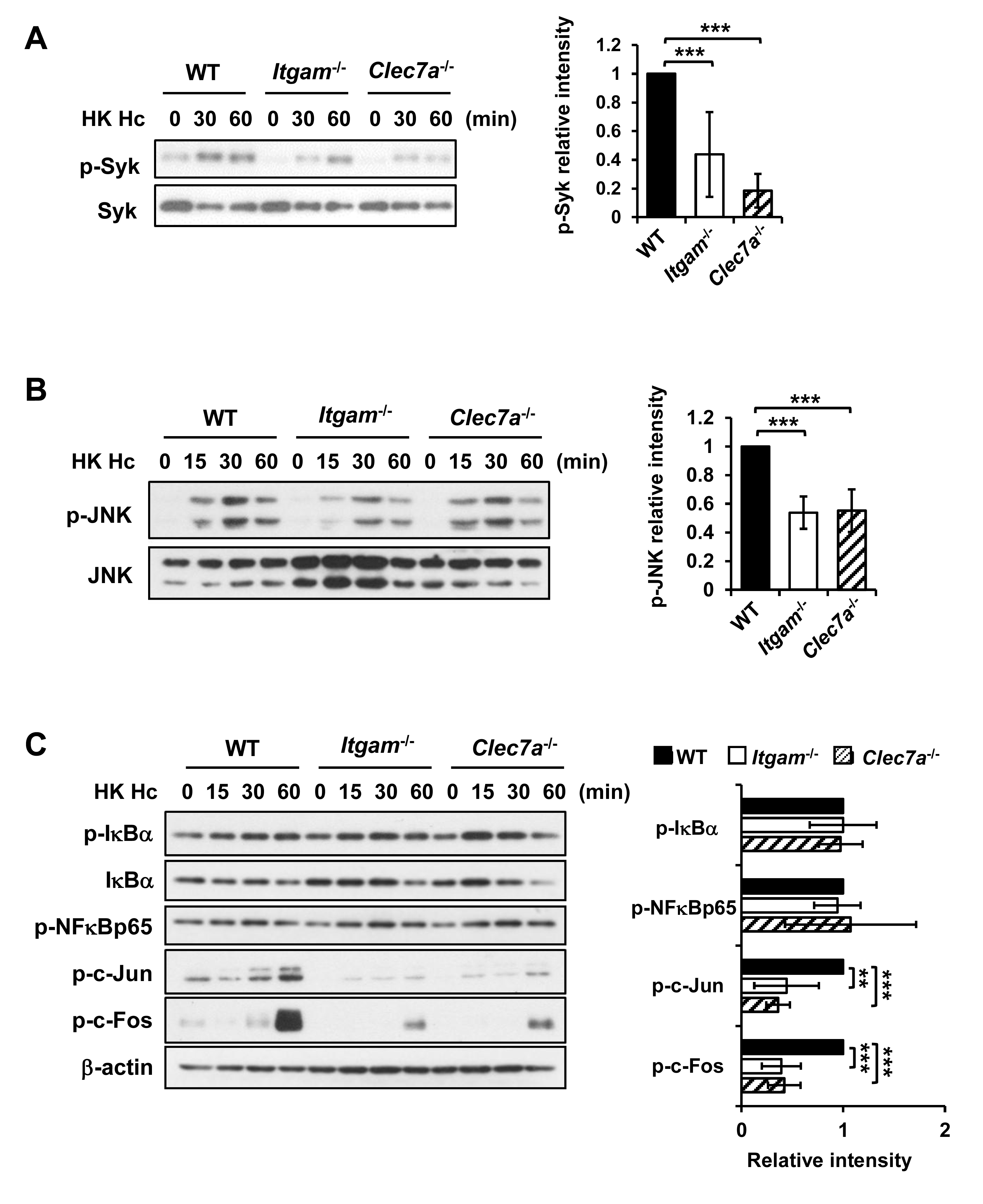

Supplement: S5 Fig — Macrophages from WT, Itgam -/-, Clec7a -/- mice were stimulated with or without (0 min) HK H. capsulatum for 15, 30 and 60 min. Cell lysates were analyzed by Western blotting for activated Syk (A), JNK (B), AP-1 and NF-κB (C). The intensity of p-Syk (A), p-JNK (B), p-IκBα, IκBα and p-NF-κBp65 at 30 min and p-c-Jun and p-c-Fos at 60 min (C) after stimulation was normalized against the corresponding internal controls. Data shown in the right panel of (A-C) are the mean ± SD of relative intensity (n = 5). ** p ≦ 0.01, *** p ≦ 0.001 [2-tailed t-test]. (TIF) [file ppat.1004985.s005.tif]

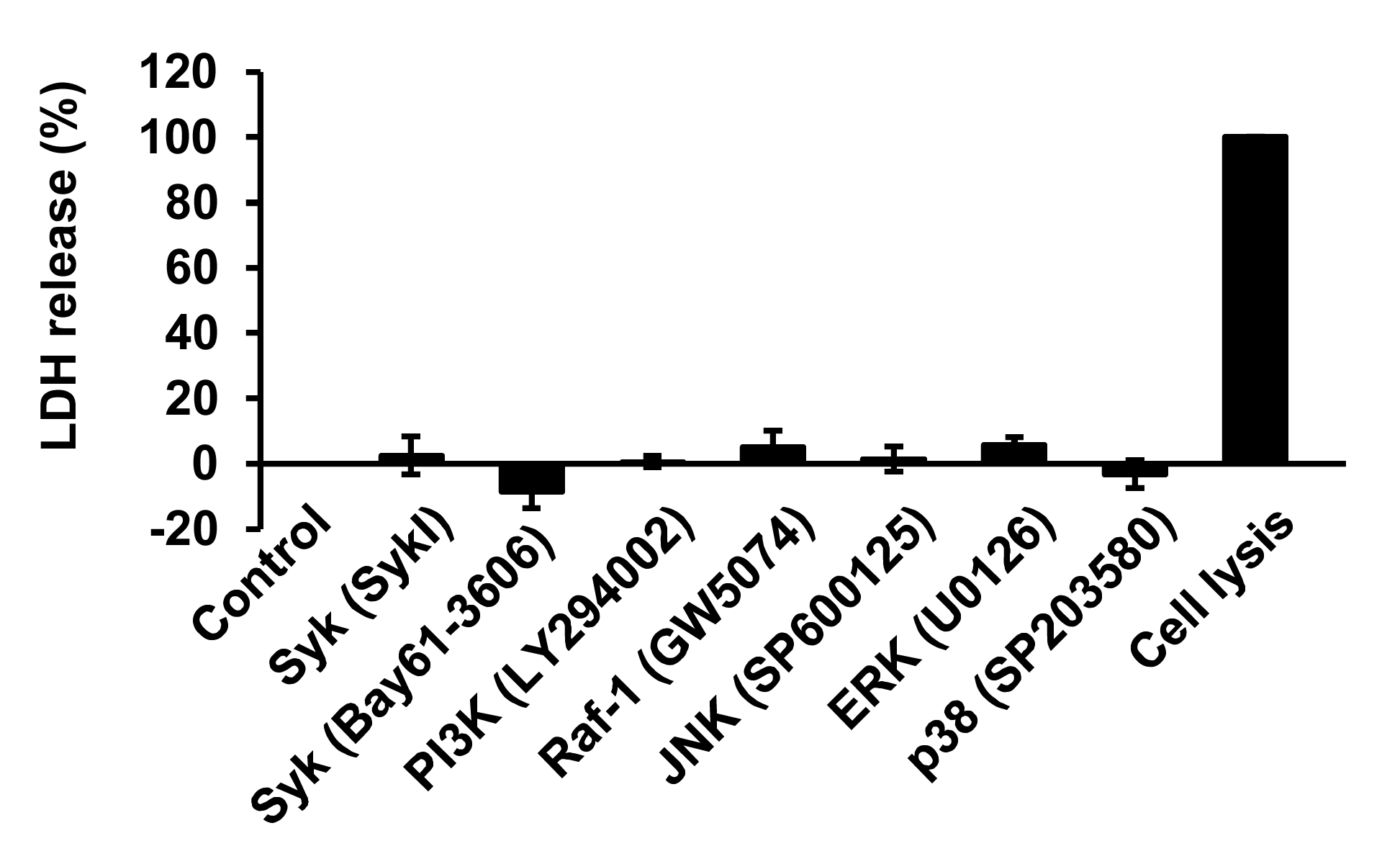

Supplement: S6 Fig — LDH release by macrophages cultured in medium containing vehicle (control) or indicated kinase inhibitors for 7 h. Data shown are the mean ± SD of the percentage of LDH release (n = 3). (TIF) [file ppat.1004985.s006.tif]

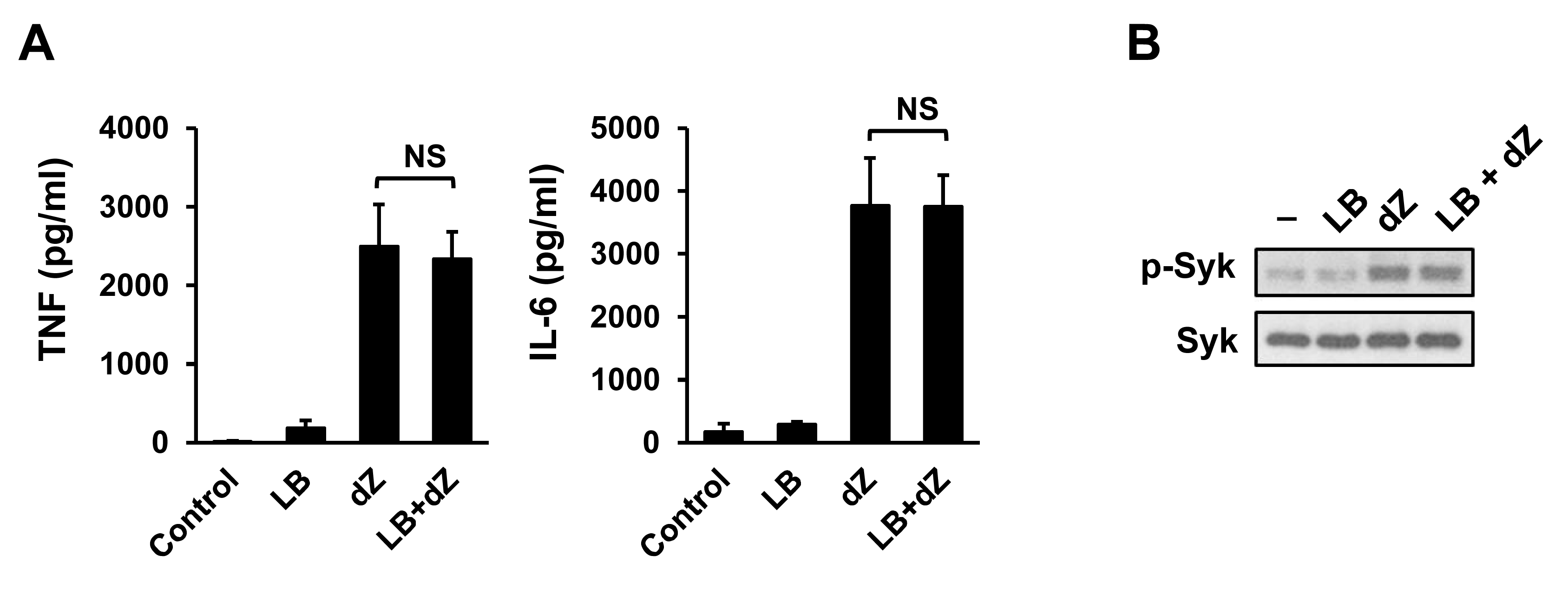

Supplement: S7 Fig — Macrophages from WT mice were stimulated with or without uncoated Latex beads (LB), depleted zymosan (dZ), or their combination for 6 h (A) or 30 min (B). (A) The concentrations of TNF and IL-6 in culture supernatants were quantified by ELISA. Mean ± SD are shown (n = 4). (B) Cell lysates were collected and analyzed by Western blotting for Syk activation. NS, not significant [2-tailed t-test]. (TIF) [file ppat.1004985.s007.tif]

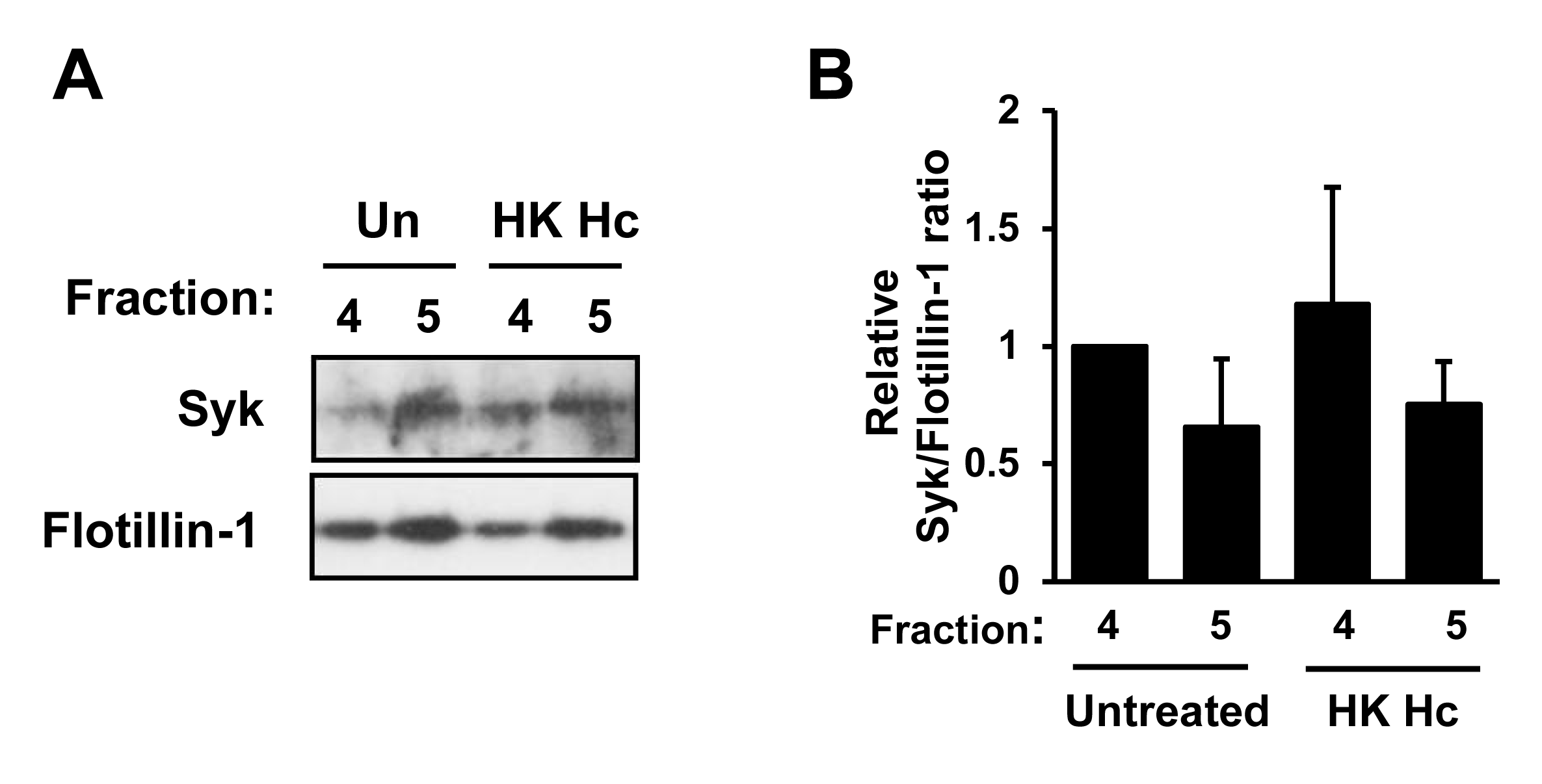

Supplement: S8 Fig — Macrophages were stimulated with or without HK H. capsulatum for 30 min. (A) Cell lysates were subjected to sucrose gradient ultracentrifugation. The presence of Syk in each fraction was analyzed by Western blotting. The blot probed with anti-flotillin-1 antibody was used to identify lipid raft fractions. (B) Bar graph shows the relative ratio of Syk to flotillin-1. That in fraction 4 of unstimulated cells was set as 1. Data presented are the mean ± SD (n = 3). (TIF) [file ppat.1004985.s008.tif]

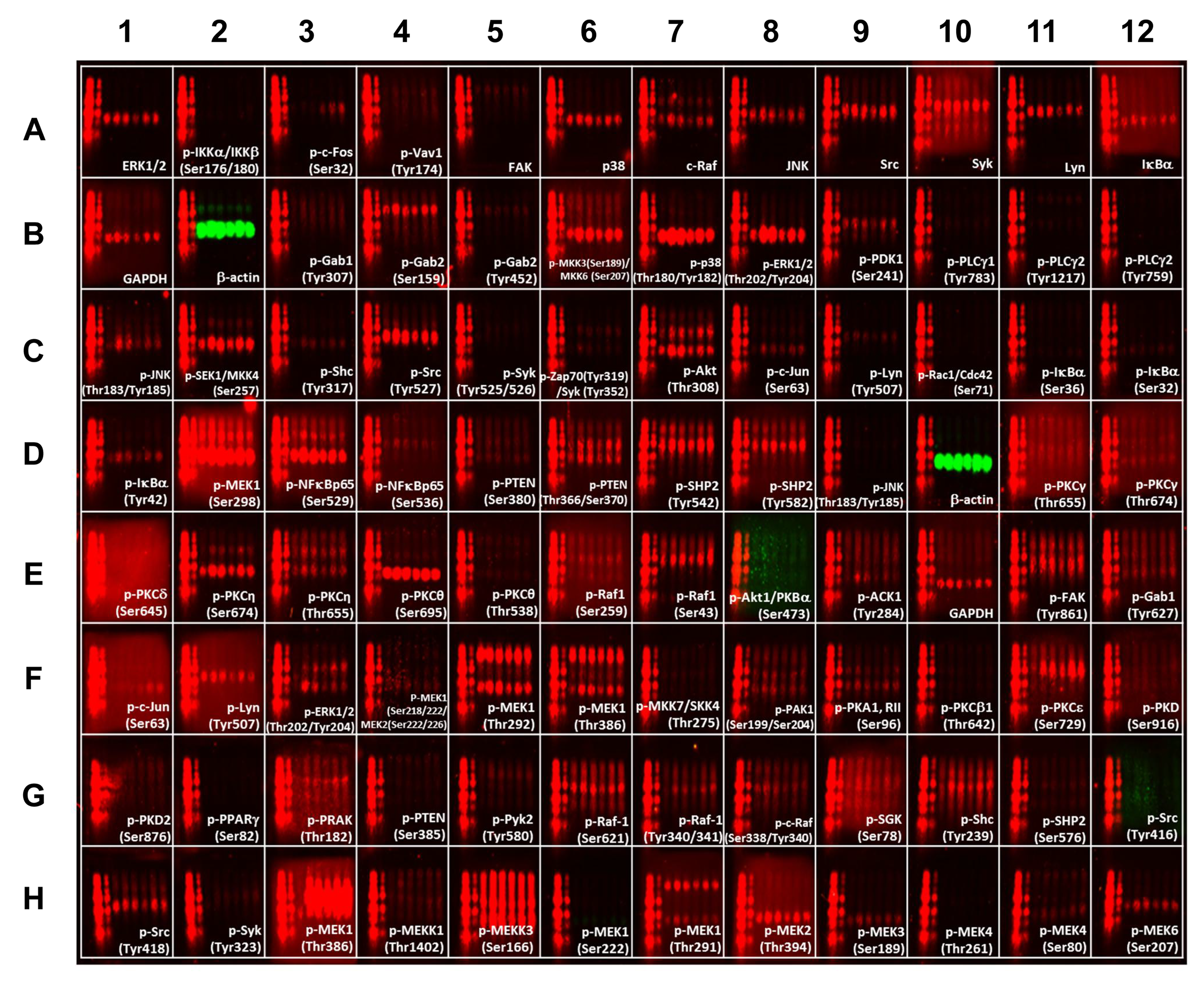

Supplement: S9 Fig — The signaling molecules in macrophages stimulated with or without HK H. capsulatum were screened by Micro-Western Array (MWA) to measure the changes in abundance of indicated proteins. The six samples printed in each well from left to right are macrophages unstimulated (0 min), and stimulated with HK H. capsulatum at a yeast-to-cell ratio of 20/1 for 15, 30, 60, 90, and 120 min. The red and green signals represent samples probed with secondary anti-rabbit and anti-mouse antibodies, respectively. The fluorochrome intensities were analyzed by Odyssey analysis software. S1 Table lists the antibodies used for blotting in each well of the 96-well array. (TIF) [file ppat.1004985.s009.tif]

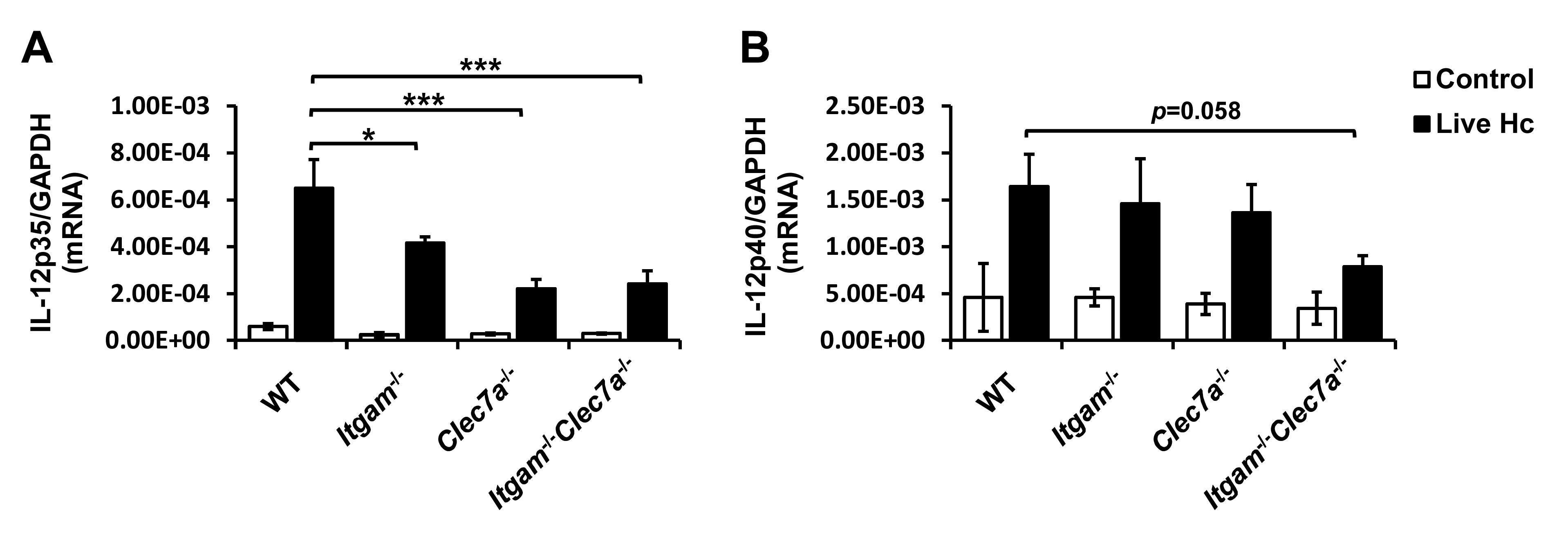

Supplement: S10 Fig — BMDCs from WT, Itgam -/-, Clec7a -/- and Itgam -/- Clec7a -/- mice were stimulated with or without live H. capsulatum (MOI = 2) for 6 h. The expression levels of IL-12p35 (A) and IL-12p40 (B) mRNA were analyzed by real-time qPCR. Data shown are the mean ± SD of relative transcript normalized against GAPDH (n = 3). * p ≦ 0.05, *** p ≦ 0.001 [one-way ANOVA with Tukey post-hoc analysis]. (TIF) [file ppat.1004985.s010.tif]

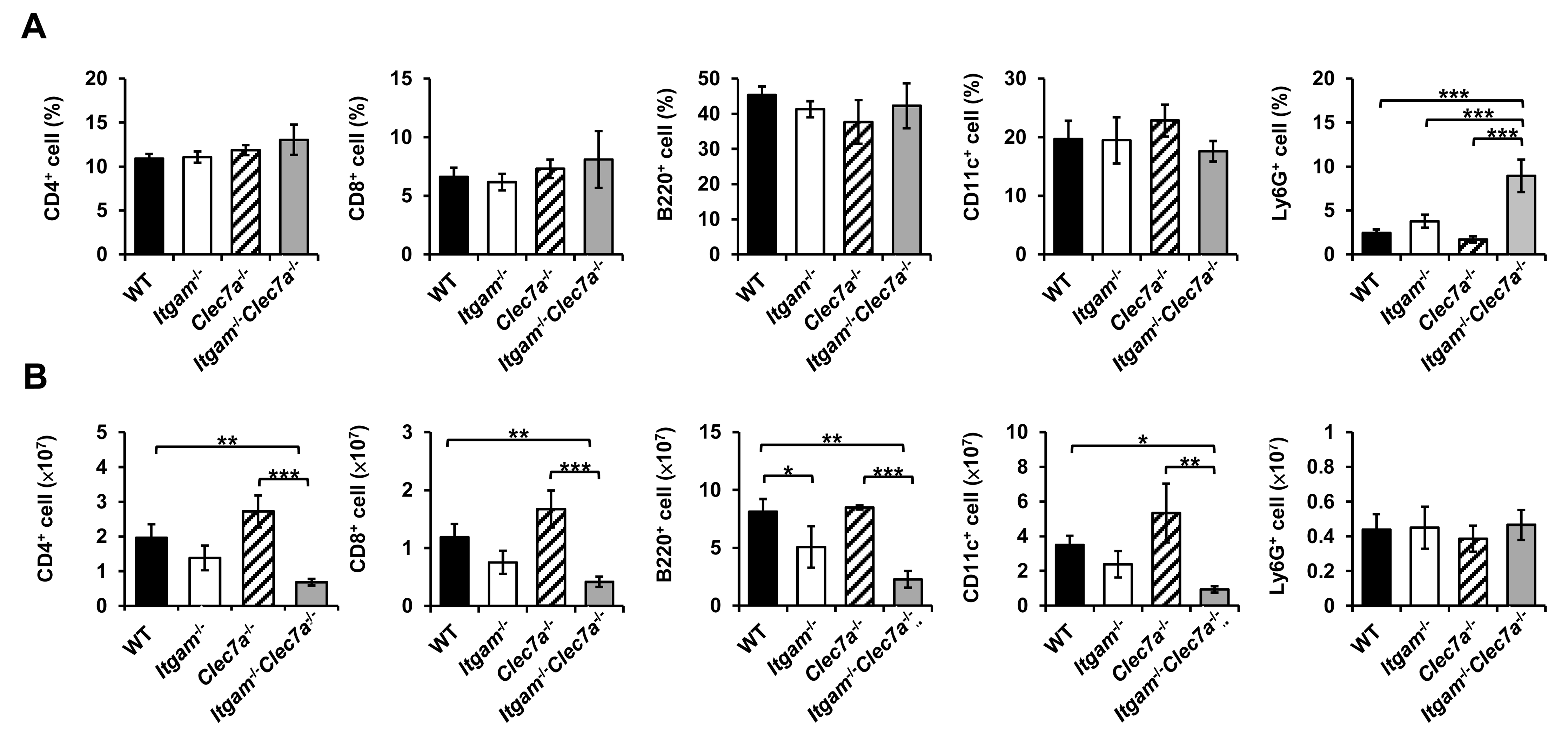

Supplement: S11 Fig — WT, Itgam -/-, Clec7a -/-, and Itgam -/- Clec7a -/- mice were infected with 5 × 10^6^ H. capsulatum intravenously. Infected mice were killed on day 9 after infection. The percentage (A) and number (B) of CD4+, CD8+, B220+, CD11c+ and Ly6G+ cells in the spleen was analyzed by flow cytometry. Mean ± SD are shown (n = 3-4). * p ≦ 0.05, ** p ≦ 0.01, *** p ≦ 0.001 [one-way ANOVA with Duncan post-hoc analysis]. (TIF) [file ppat.1004985.s011.tif]

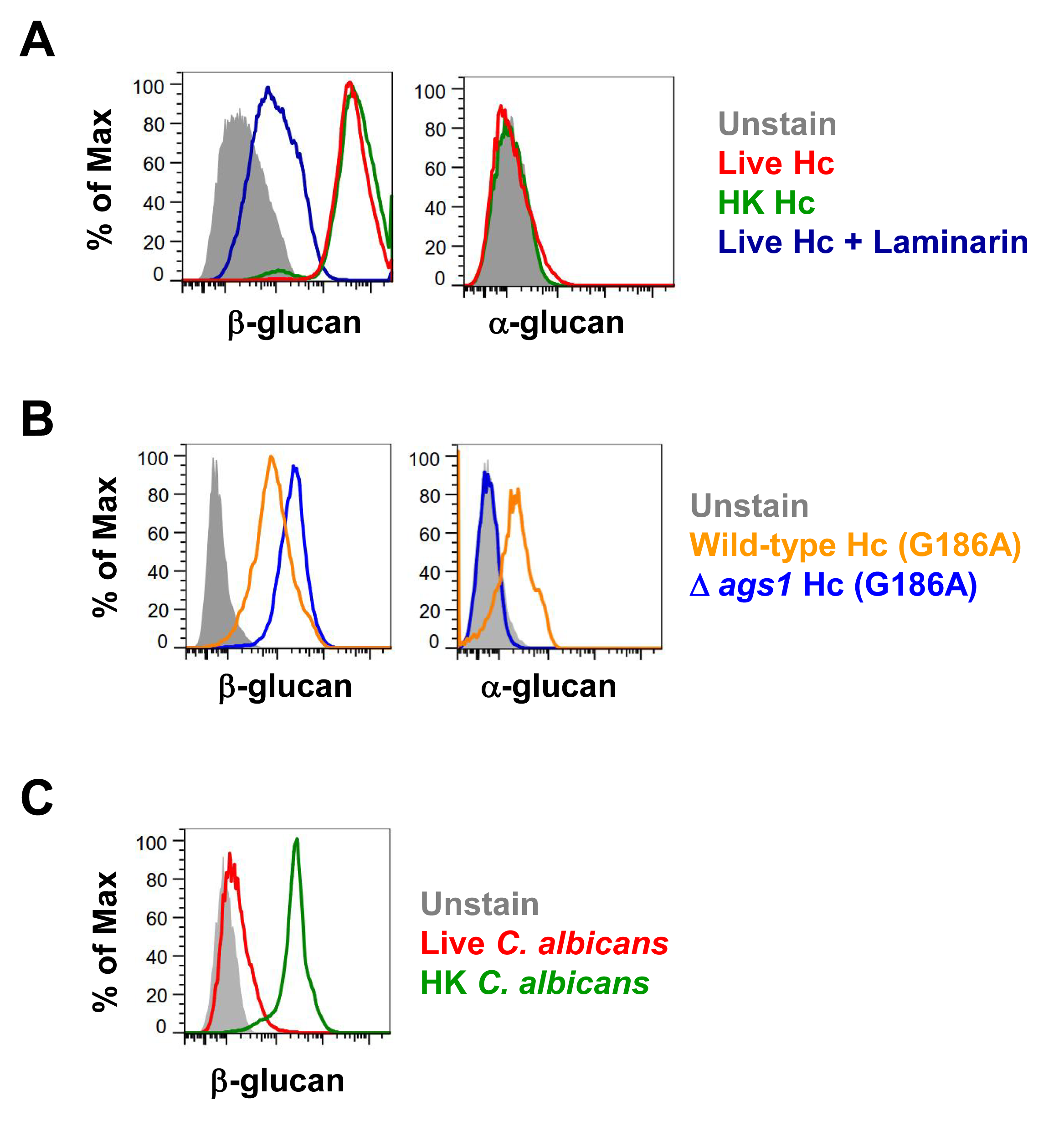

Supplement: S12 Fig — (A) H. capsulatum strain 505 has β-glucan exposed and lacks α-glucan expression on the yeast cell wall. Live or HK H. capsulatum was stained with anti-α-glucan or anti-β-glucan antibody in the presence or absence of laminarin and analyzed by flow cytometry. (B) H. capsulatum strain G186A expressing α-glucan masks β-glucan on the yeast cell wall. Viable wild-type or ags1-null mutant H. capsulatum strain G186A were stained for surface expression of α-(1,3)-glucan and β-(1,3)-glucan and analyzed by flow cytometry. (C) Heat treatment exposes β-glucan on the surface of C. albicans. Viable or HK C. albicans strain SC5314 were stained for surface β-(1,3)-glucan and analyzed by flow cytometry. (TIF) [file ppat.1004985.s012.tif]

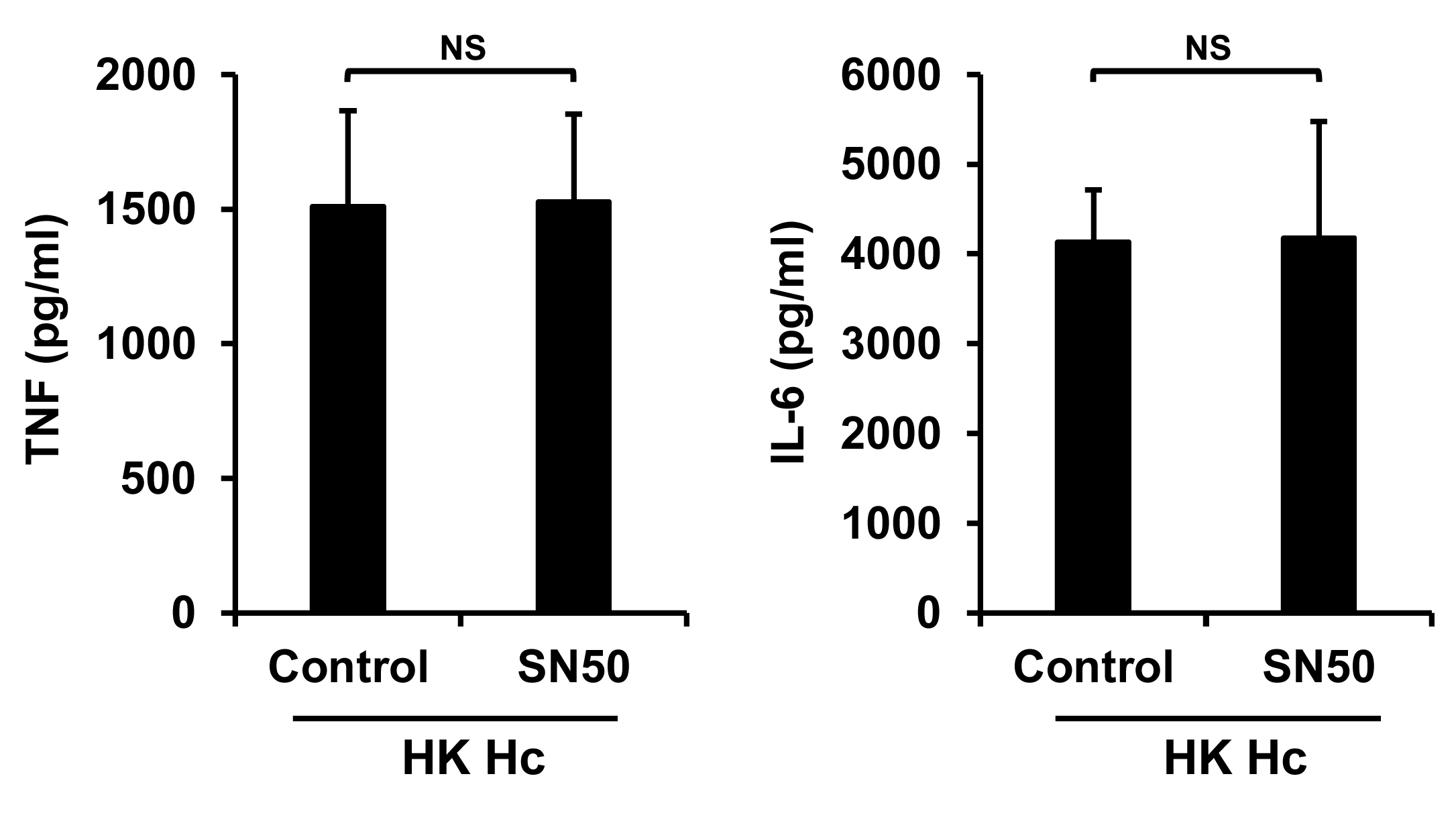

Supplement: S13 Fig — Macrophages from WT mice were treated with vehicle (control) or NF-κB inhibitor (SN50, 50 μg/ml) for 1 h prior to stimulation with HK H. capsulatum. Culture supernatants were collected 6 h later and the levels of TNF and IL-6 were quantified by ELISA. Mean ± SD are shown (n = 5). NS, not significant [2-tailed t-test]. (TIF) [file ppat.1004985.s013.tif]

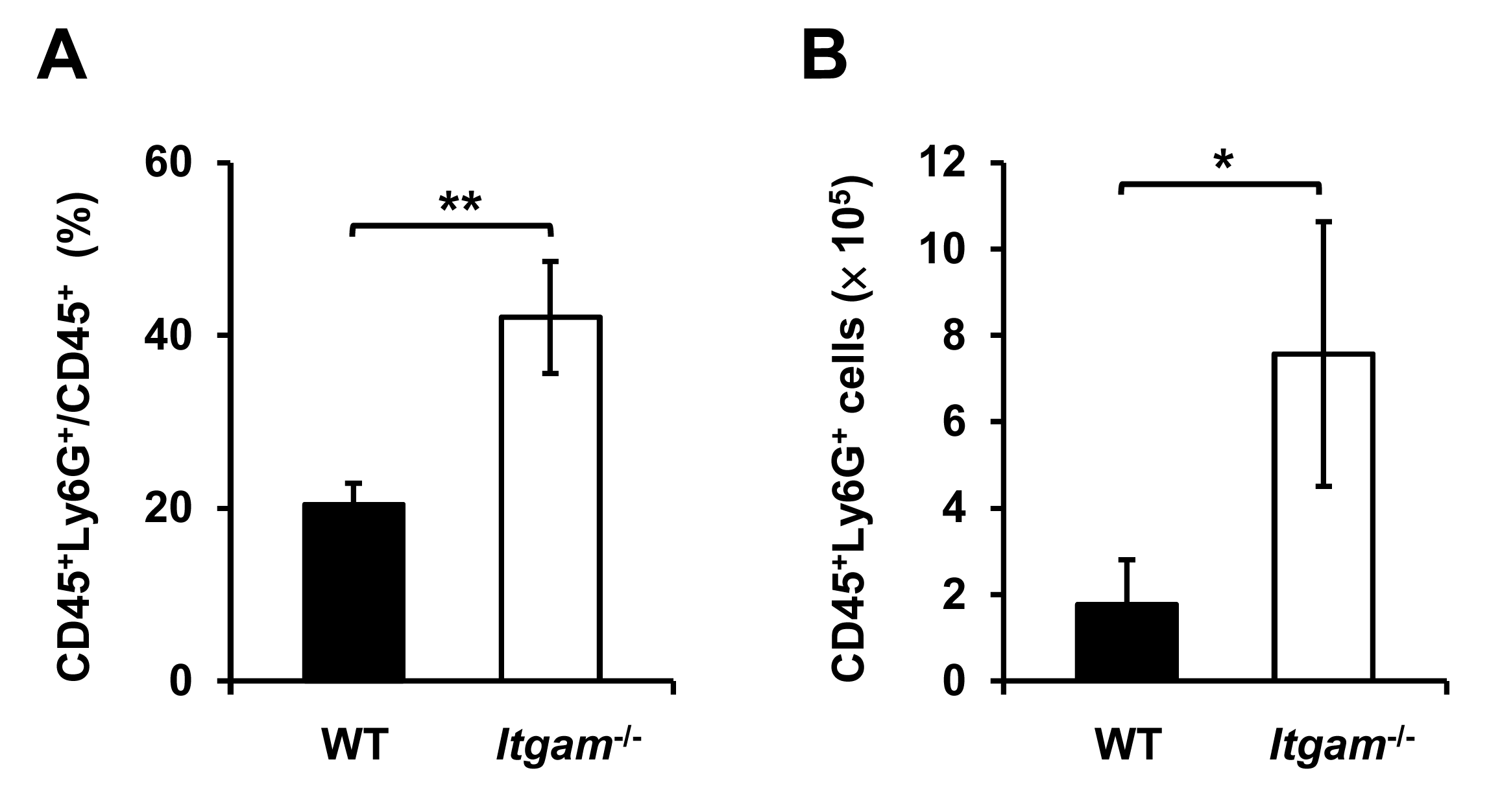

Supplement: S14 Fig — WT and Itgam -/- mice were infected with 2.5 × 10^5^ H. capsulatum intratracheally. Mice were killed on day 7 post-infection. Leukocytes were isolated from lung homogenates and analyzed by flow cytometry after staining with surface CD45 and Ly6G. (A) Percentage of CD45+Ly6G+ cells in CD45+ population. (B) Number of CD45+Ly6G+ cells in the lungs. Mean ± SD are shown (n =3). * p ≦ 0.05, ** p ≦ 0.01 [2-tailed t-test]. (TIF) [file ppat.1004985.s014.tif]
