# Supplementary material for: CR3 and Dectin-1 Collaborate in Macrophage Cytokine Response through Association on Lipid Rafts and Activation of Syk-JNK-AP-1 Pathway
Source: PLoS Pathog. 2015 Jul 1;11(7):e1004985. doi: 10.1371/journal.ppat.1004985 (PMC4488469; doi:10.1371/journal.ppat.1004985)
Supplement: S1 Table — (DOCX) [file ppat.1004985.s015.docx]

| **MWA position** | **Antibody** | **Molecular weight** | **Host species** | **MWA position** | **Antibody** | **Molecular weight** | **Host species** |
| --- | --- | --- | --- | --- | --- | --- | --- |
| **A1** | **p44/42 MAPK (ERK1/2), clone 137F5** | **42, 44 kDa** | **Rabbit** | **E1** | **phospho-PKCδ (Ser645)** | **78 kDa** | **Rabbit** |
| **A2** | **Phospho-IKKα/β (Ser176/180), clone 16A6** | **85, 87 kDa** | **Rabbit** | **E2** | **phospho-PKCη (Ser674)** | **80 kDa** | **Rabbit** |
| **A3** | **phospho-c-Fos (Ser32), clone D82C12** | **62 kDa** | **Rabbit** | **E3** | **phospho-PKCη (Thr655)** | **80 kDa** | **Rabbit** |
| **A4** | **phospho-Vav1 (Tyr174), clone EP510Y** | **101 kDa** | **Rabbit** | **E4** | **phospho-PKCθ (Ser695)** | **80 kDa** | **Rabbit** |
| **A5** | **FAK** | **125 kDa** | **Rabbit** | **E5** | **phospho-PKCθ (Thr538)** | **80 kDa** | **Rabbit** |
| **A6** | **p38α MAPK, clone 7D6** | **40 kDa** | **Rabbit** | **E6** | **phospho-Raf1 (Ser259)** | **74 kDa** | **Rabbit** |
| **A7** | **Raf-(C)** | **65-75 kDa** | **Rabbit** | **E7** | **phospho-Raf1 (Ser43)** | **70 kDa** | **Rabbit** |
| **A8** | **SAPK/JNK, clone 56G8** | **46, 54 kDa** | **Rabbit** | **E8** | **phospho-Akt1/PKBα (Ser473), clone 11E6** | **60 kDa** | **Mouse** |
| **A9** | **Src, clone 36D10** | **60 kDa** | **Rabbit** | **E9** | **phospho-ACK1 (Tyr284)** | **114 kDa** | **Rabbit** |
| **A10** | **Syk** | **72 kDa** | **Rabbit** | **E10** | **GAPDH** | **35 kDa** | **Rabbit** |
| **A11** | **Lyn, clone C13F9** | **56 kDa** | **Rabbit** | **E11** | **phospho-FAK (Tyr861)** | **125 kDa** | **Rabbit** |
| **A12** | **IκB-α** | **37 kDa** | **Rabbit** | **E12** | **phospho-Gab1 (Tyr627)** | **105 kDa** | **Rabbit** |
| **B1** | **GAPDH** | **35 kDa** | **Rabbit** | **F1** | **phospho-Jun(-c) (Ser63), clone Y172** | **36-42 kDa** | **Rabbit** |
| **B2** | **Actin** | **43 kDa** | **Mouse** | **F2** | **phospho-Lyn (Tyr507)** | **53, 56 kDa** | **Rabbit** |
| **B3** | **Phospho-Gab1 (Tyr307)** | **115 kDa** | **Rabbit** | **F3** | **phospho-MAP Kinase1/2 (Erk1/2)** | **42, 44 kDa** | **Rabbit** |
| **B4** | **Phospho-Gab2 (Ser159)** | **98 kDa** | **Rabbit** | **F4** | **phospho-MEK1 (Ser218/222)/MEK2 (Ser222/226)** | **45 kDa** | **Rabbit** |
| **B5** | **Phospho-Gab2 (Tyr452), clone C33G1** | **98 kDa** | **Rabbit** | **F5** | **phospho-MEK1 (Thr292)** | **44 kDa** | **Rabbit** |
| **B6** | **Phospho-MKK3 (Ser189)/MKK6 (Ser207), clone 22A8** | **40, 41 kDa** | **Rabbit** | **F6** | **phospho-MEK1 (Thr386)** | **44 kDa** | **Rabbit** |
| **B7** | **Phospho-p38 MAPK (Thr180/Tyr182), clone D3F9** | **43 kDa** | **Rabbit** | **F7** | **phospho-MKK7/SKK4 (Thr275)** | **47 kDa** | **Rabbit** |
| **B8** | **Phospho-p44/42 MAPK (Erk1/2) (Thr202/Tyr204), clone D13.14.4E** | **42, 44 kDa** | **Rabbit** | **F8** | **phospho-PAK1 (Ser199/Ser204)** | **70 kDa** | **Rabbit** |
| **B9** | **Phospho-PDK1 (Ser241), clone C49H2** | **58-68 kDa** | **Rabbit** | **F9** | **phospho-PKA, RII (Ser96)** | **54 kDa** | **Rabbit** |
| **B10** | **Phospho-PLCγ1 (Tyr783)** | **155 kDa** | **Rabbit** | **F10** | **phospho-PKCβ I (Thr642)** | **80 kDa** | **Rabbit** |
| **B11** | **Phospho-PLCγ2 (Tyr1217)** | **150 kDa** | **Rabbit** | **F11** | **phospho-PKCε (Ser729)** | **95 kDa** | **Rabbit** |
| **B12** | **Phospho-PLCγ2 (Tyr759)** | **150 kDa** | **Rabbit** | **F12** | **phospho-PKD (Ser916)** | **105-110 kDa** | **Rabbit** |
| **C1** | **Phospho-SAPK/JNK (Thr183/Tyr185), clone 81E11** | **46, 54 kDa** | **Rabbit** | **G1** | **phospho-PKD2 (Ser876)** | **105 kDa** | **Rabbit** |
| **C2** | **Phospho-SEK1/MKK4 (Ser257), clone C36C11** | **44 kDa** | **Rabbit** | **G2** | **phospho-PPARγ (Ser82), clone AW504** | **55, 60 kDa** | **Rabbit** |
| **C3** | **Phospho-Shc (Tyr317)** | **50, 55, 70 kDa** | **Rabbit** | **G3** | **phospho-PRAK (Thr182)** | **54 kDa** | **Rabbit** |
| **C4** | **Phospho-Src (Tyr527)** | **60 kDa** | **Rabbit** | **G4** | **phospho-PTEN (Ser385)** | **58 kDa** | **Rabbit** |
| **C5** | **Phospho-Syk (Tyr525/526), clone C87C1** | **72 kDa** | **Rabbit** | **G5** | **phospho-Pyk2 (Tyr580)** | **115 kDa** | **Rabbit** |
| **C6** | **Phospho-Zap-70 (Tyr319)/Syk (Tyr352), clone 65E4** | **70, 72 kDa** | **Rabbit** | **G6** | **phospho-Raf-1 (Ser621)** | **74 kDa** | **Rabbit** |
| **C7** | **Phospho-Akt (Thr308)** | **60 kDa** | **Rabbit** | **G7** | **phospho-Raf-1 (Tyr340/Tyr341)** | **74 kDa** | **Rabbit** |
| **C8** | **Phospho-Jun(-c) (Ser63), clone 54B3** | **48 kDa** | **Rabbit** | **G8** | **phospho-Raf(-c) (Ser338/Tyr340)** | **74 kDa** | **Rabbit** |
| **C9** | **Phospho-Lyn (Tyr507)** | **53, 56 kDa** | **Rabbit** | **G9** | **phospho-SGK (Ser78)** | **48 kDa** | **Rabbit** |
| **C10** | **phospho-Rac1/cdc42 (Ser71)** | **28 kDa** | **Rabbit** | **G10** | **phospho-SHC (Tyr239)** | **42, 52, 67 kDa** | **Rabbit** |
| **C11** | **phospho-IκB-α (Ser36)** | **40 kDa** | **Rabbit** | **G11** | **phospho-SHP-2 (Ser576)** | **68 kDa** | **Rabbit** |
| **C12** | **phospho-IκB-α (Ser32)** | **40 kDa** | **Rabbit** | **G12** | **phospho-Src (Tyr416), clone 9A6** | **60 kDa** | **Mouse** |
| **D1** | **phospho-IκB-α (Tyr42)** | **37 kDa** | **Rabbit** | **H1** | **phospho-Src (Tyr418)** | **60 kDa** | **Rabbit** |
| **D2** | **Phospho-MEK1 (Ser298)** | **45 kDa** | **Rabbit** | **H2** | **phospho-Syk (Tyr323)** | **72 kDa** | **Rabbit** |
| **D3** | **Phospho-NF-κB p65 (Ser529)** | **65 kDa** | **Rabbit** | **H3** | **phospho-MEK-1 (Thr386)** | **45 kDa** | **Rabbit** |
| **D4** | **Phospho-NF-κB p65 (Ser536)** | **70 kDa** | **Rabbit** | **H4** | **phospho-MEK kinase-1 (Thr1402)** | **195 kDa** | **Rabbit** |
| **D5** | **Phospho-PTEN (Ser380)** | **54 kDa** | **Rabbit** | **H5** | **phospho-MEK kinase-3 (Ser166)** | **71 kDa** | **Rabbit** |
| **D6** | **Phospho-PTEN (Thr366/Ser370)** | **54 kDa** | **Rabbit** | **H6** | **phospho-MEK-1 (Ser222)** | **45 kDa** | **Rabbit** |
| **D7** | **Phospho-SHP-2 (Tyr542)** | **72 kDa** | **Rabbit** | **H7** | **phospho-MEK-1 (Thr291)** | **45 kDa** | **Rabbit** |
| **D8** | **Phospho-SHP-2 (Tyr582)** | **72 kDa** | **Rabbit** | **H8** | **phospho-MEK-2 (Thr394)** | **47 kDa** | **Rabbit** |
| **D9** | **phospho-JNK (Thr183/Tyr185, Thr221/Tyr223)** | **42 kDa** | **Rabbit** | **H9** | **phospho-MEK-3 (Ser189)** | **40 kDa** | **Rabbit** |
| **D10** | **Actin** | **43 kDa** | **Mouse** | **H10** | **phospho-MEK-4 (Thr261)** | **45 kDa** | **Rabbit** |
| **D11** | **phospho-PKCγ (Thr655)** | **80 kDa** | **Rabbit** | **H11** | **phospho-MEK-4 (Ser80)** | **45 kDa** | **Rabbit** |
| **D12** | **phospho-PKCγ (Thr674)** | **80 kDa** | **Rabbit** | **H12** | **phospho-MEK-6 (Ser207)** | **37 kDa** | **Rabbit** |
